# Do computerised clinical decision support systems for prescribing change practice? A systematic review of the literature (1990-2007)

**DOI:** 10.1186/1472-6963-9-154

**Published:** 2009-08-28

**Authors:** Sallie-Anne Pearson, Annette Moxey, Jane Robertson, Isla Hains, Margaret Williamson, James Reeve, David Newby

**Affiliations:** 1 UNSW Cancer Research Centre, University of New South Wales and Prince of Wales Clinical School, Sydney, Australia; 2 Discipline of Clinical Pharmacology, School of Medicine and Public Health, The University of Newcastle, Australia; 3 National Prescribing Service Limited, Sydney, Australia

## Abstract

**Background:**

Computerised clinical decision support systems (CDSSs) are used widely to improve quality of care and patient outcomes. This systematic review evaluated the impact of CDSSs in targeting specific aspects of prescribing, namely initiating, monitoring and stopping therapy. We also examined the influence of clinical setting (institutional vs ambulatory care), system- or user-initiation of CDSS, multi-faceted vs stand alone CDSS interventions and clinical target on practice changes in line with the intent of the CDSS.

**Methods:**

We searched Medline, Embase and PsychINFO for publications from 1990-2007 detailing CDSS prescribing interventions. Pairs of independent reviewers extracted the key features and prescribing outcomes of methodologically adequate studies (experiments and strong quasi-experiments).

**Results:**

56 studies met our inclusion criteria, 38 addressing initiating, 23 monitoring and three stopping therapy. At the time of initiating therapy, CDSSs appear to be somewhat more effective after, rather than before, drug selection has occurred (7/12 versus 12/26 studies reporting statistically significant improvements in favour of CDSSs on = 50% of prescribing outcomes reported). CDSSs also appeared to be effective for monitoring therapy, particularly using laboratory test reminders (4/7 studies reporting significant improvements in favour of CDSSs on the majority of prescribing outcomes). None of the studies addressing stopping therapy demonstrated impacts in favour of CDSSs over comparators. The most consistently effective approaches used system-initiated advice to fine-tune existing therapy by making recommendations to improve patient safety, adjust the dose, duration or form of prescribed drugs or increase the laboratory testing rates for patients on long-term therapy. CDSSs appeared to perform better in institutional compared to ambulatory settings and when decision support was initiated automatically by the system as opposed to user initiation. CDSSs implemented with other strategies such as education were no more successful in improving prescribing than stand alone interventions. Cardiovascular disease was the most studied clinical target but few studies demonstrated significant improvements on the majority of prescribing outcomes.

**Conclusion:**

Our understanding of CDSS impacts on specific aspects of the prescribing process remains relatively limited. Future implementation should build on effective approaches including the use of system-initiated advice to address safety issues and improve the monitoring of therapy.

## Background

Computerised clinical decision support systems (CDSSs) are espoused as powerful tools for influencing health care provider performance to improve quality of care and patient outcomes. As such, proprietary and locally developed systems have been used to target provider behaviour across a range of clinical circumstances including preventive, acute and chronic care and to target specific test ordering and prescribing practices.

In their simplest form CDSSs provide narrative information requiring further processing and analysis by providers before clinical decisions are made. However, CDSSs have become increasingly sophisticated by matching patient characteristics with computerised knowledge bases and using algorithms to generate patient-specific assessments or treatment recommendations. [1] CDSSs support a range of prescribing practice activities relating broadly to initiating therapy, including the judicious selection of drug treatments, adhering to best practice care and checking allergies and drug interactions. In addition, CDSSs can target other aspects of the prescribing process, namely monitoring (with dose adjustments) and stopping or tapering therapy.

The most comprehensive systematic reviews in the field [1,2] established the benefits of CDSSs in changing practitioner performance and to a lesser extent, patient outcomes. Garg et al [1] reviewed 100 randomised and non-randomised studies of CDSSs across a broad range of clinical targets including diagnosis, prevention, disease management, drug dosing and drug prescribing while the Kawamoto et al [2] review was restricted to randomised controlled trials (RCTs) but assessed the impact of both electronic and non-electronic systems. Both reviews also examined system and organisational features predicting the success of decision support.

Notably, improved CDSS performance was associated with systems prompting providers automatically (system-initiated) rather than requiring providers to activate the decision support themselves (user-initiated).[1] Kawamoto et al also reported the benefits of computerised over paper-based systems and those providing advice within the clinical workflow and at the time and location of decision-making.[2] A subsequent review has also demonstrated systems implemented in hospital practice are more effective in improving outcomes than those targeting chronic disease in ambulatory care.[3]

Other systematic reviews have taken a narrower prescribing focus and demonstrate CDSSs can reduce toxic drug levels and time to achieving therapeutic control [4,5]; reduce medication errors [6,7]; change prescribing in accordance with guideline recommendations and increase diagnostic testing. [8]

In a more recent systematic review, Mollon et al[9] examined the effects of CDSSs on prescribing practices and evaluated whether particular system features could predict successful implementation, provider behaviour change or improved patient outcomes. The review was restricted to RCTs of computer generated advice (delivered in electronic or paper-based formats) and targeted decisions presented before drug therapies were chosen or directly after drug selection had taken place. The authors noted few high quality studies showed improvement in patient outcomes and there was insufficient detail on system features in the original studies to inform future CDSS design and implementation. None of the reviews conducted to date have examined the effects of CDSSs across all aspects of the prescribing process nor addressed clinical targets and how they may relate to the relative success of CDSSs.

Providers' information needs are likely to vary across clinical targets and the therapeutic process (initiating, monitoring and stopping therapy), and it is highly likely that some aspects of prescribing practice are more amenable to change than others. Previous research has suggested physicians are more reluctant to change practice if advice is perceived as threatening to professional autonomy.[10,11] Thus, recommendations attempting to influence initial therapeutic choices or suggestions to stop long-standing treatment may be more difficult to change than attempts to flag safety issues or to fine-tune existing therapy. Further, prescribing involves a complex set of behaviours and there is strong evidence demonstrating the benefits of multi-faceted, compared with single strategy approaches to prescriber behaviour change. [12-14]

Therefore, the objective of this systematic review is to examine the impact of CDSSs in targeting specific aspects of prescribing, namely initiating treatment (before and after drug selection has taken place), monitoring patients on existing therapy and stopping treatment. Further, within each domain, we will explore whether some of the key technical and organisational features shown to be effective in previous CDSS reviews change practice in line with the intent of the CDSS, namely system versus user-initiated advice, CDSSs implemented in institutional settings versus ambulatory care, and multi-faceted versus stand alone CDSS interventions. Finally, we will explore the clinical areas targeted in the CDSS interventions.

## Methods

### Hypotheses Tested

We hypothesised that CDSSs for prescribing, where advice is generated and delivered electronically, are more likely to change prescribing practice in line with the intent of the CDSS when decision support is:

• Provided to fine-tune existing therapy compared with recommendations to influence initial drug choices or cease long-standing therapy.

Within the various prescribing domains we also hypothesised that CDSS is more likely to change prescribing practice in line with the intent of the CDSS when decision support is:

• Implemented in institutional (inpatient) settings compared with ambulatory care

• System-initiated compared with user-initiated

• Implemented with other intervention strategies compared with implemented alone.

In addition, we were interested in exploring the clinical areas targeted in CDSS interventions. While it is unlikely there would be sufficient studies to test a formal hypothesis relating to clinical target, we aimed to document the clinical targets within prescribing domain and establish if computerised CDSS is more effective in specific clinical areas.

### Studies Eligible for Review

We included English-language studies, published since 1990, reporting RCTs and strong quasi-experiments (non-randomised studies with comparison groups or interrupted time series designs with or without comparison groups). We stipulated the studies had to: compare the performance of computerised CDSS to routine care and/or paper-based decision support; provide information applicable to a specific patient that is reviewed by a provider at the time of prescribing (e.g. provide advice to prescribe a particular drug, to monitor a drug or adjust the dose, or to perform laboratory tests related to safe prescribing); generate and deliver information to the provider in electronic formats; and report data on at least one outcome relating to initiating, monitoring or stopping therapy (Table 1).

**Table 1:** Definitions and examples of initiating, monitoring and stopping therapy

**Initiating Therapy**	Provides suggestions on which drug to prescribe (or not to prescribe) when a new course of therapy is started.
	Examples:
	Prescribe a cholesterol lowering medication for patients with LDL > 130 mg/dL
	Administer influenza vaccination
	Do not prescribe long-acting benzodiazepines for elderly patients
	Suggestions may be presented to the physician *before choosing*a drug (e.g. patient's cholesterol level triggers an alert recommending the use of statins) or *after making a prescribing choice*(e.g. drug interaction alert may prompt a change in medicine selected).
**Monitoring**	Provides suggestions for patients on continuing drug therapy (i.e. past the initial prescribing decision).
	Examples:
	Increase or decrease dose for patients on existing therapy (e.g. inhaler dose for the prevention of asthma)
	Therapeutic drug monitoring (e.g. laboratory tests to avoid drug toxicity)
	Titration to target (e.g. INR range and warfarin dosing)

**Stopping Therapy**	Guidance suggesting that a particular medication could be stopped or doses tapered with a view to stopping.
	Examples:
	Discontinuation of long-acting benzodiazepines in elderly patients receiving this drug

Studies were excluded if the CDSS targeted only medical students, pharmacists or nurses, interventions were based around hypothetical scenarios rather than actual clinical practice, and the advice provided by CDSS was feedback about groups of patients (e.g. audit activities). Further studies not undertaking statistical analyses and/or reporting only patient outcomes (e.g. quality of life, satisfaction with care, greater physical activity levels) or cost data were also excluded. A list of excluded studies is available on request from the authors.

### Study Identification

We searched Medline (1990 - November Week 3, 2007), PreMedline (30 November, 2007), Embase (1990 - Week 47, 2007), CINAHL (1990 - November Week 4, 2007) and PsycINFO (1990 - November Week 4, 2007). We combined keywords and subject headings to identify computer-based decision support (e.g. *decision support systems clinical, decision making computer assisted*), medicines use (e.g. *prescription drug, drug utilization*) and medical practice (e.g. *physicians practice patterns, medical practice*). We also searched INSPEC (November 2007) and the Cochrane Database of Systematic Reviews (November 2007) including reviews and protocols published under the Effective Practice and Organisation of Care Group (EPOC). Finally, we hand-searched the reference lists of retrieved articles and reviews. Additional File 1 details the full search strategy.

Pairs of reviewers evaluated independently the study titles and abstracts identified in the search. Full-text articles were retrieved if any reviewer considered a citation potentially relevant. The studies were further assessed for their relevance using a screening tool based on study design, intervention target and type of intervention.

### Data Extraction

Studies deemed eligible for review underwent data extraction by pairs of reviewers independent of one another (AM and IH, IH and SP). Disagreements were resolved by discussion to reach consensus. We developed a data extraction instrument based on checklists used in previous systematic reviews [1,13-15] but refined for the specific aims of this study. We extracted the following information: study objectives, clinical setting (ambulatory or institutional care), details of decision support intervention (e.g. system- or user-initiated, multi-faceted or CDSS alone, clinical target). Given the lack of uniformity in relation to terminology about prompts, alerts and reminders we extracted details as they were reported in the manuscripts. We recorded details of the study design (including the unit of intervention, comparison groups or measures, group assignment, statistical analyses), participant numbers, outcomes of interest and results pertaining to these outcomes.

### Quality Assessment

Two reviewers (JR and DN) rated experimental and quasi-experimental studies for methodological quality on a 10-point scale consisting of five potential sources of bias (as described by Garg and colleagues [1]). The items used to assess study quality are described along with the outcomes of this assessment in the results section. We also noted whether statistical analyses were adjusted for clustering (this was not part of the Garg assessment form).

### Reporting

Due to heterogeneity in study methodology, comparison groups, setting, intervention targets, and outcomes, we could not use traditional meta-analytic approaches to combine individual study results.

The studies in this review differed substantially in the type and number of outcomes reported to measure intervention impacts. In particular, clinical outcomes are not reported across all studies, most likely due to the short-term nature of the trials. This review only reports on the impact of CDSSs on measures relating to prescribing and includes laboratory or monitoring tests relevant for the safe and appropriate use of particular medicines. While these are intermediate outcomes, where studies have demonstrated the use of a particular drug leads to improvements in important patient outcomes, changes in prescribing consistent with this evidence are likely to be a reasonable surrogate for patient outcomes. We do, however, detail additional outcomes and provide a more extensive description of our research methods in a related report entitled 'Improving the uptake of evidence-based drug information and decision support' .

Outcomes in this review are summarised separately for each study and coded according to the following scheme:

• "+ (NS)": intervention favoured CDSS (prescribing was more consistent with the intentions of the CDSS) but was not statistically significant;

• "- (NS)": intervention favoured the comparison group (prescribing of comparison groups was more consistent with the intentions of the CDSS) but was not significant;

• "**++**": intervention favoured CDSS (prescribing was more consistent with the intentions of the CDSS) and was statistically significant;

• "**- -**": intervention favoured the comparison group (prescribing of comparison groups was more consistent with the intentions of the CDSS) and was statistically significant;

• "0": no difference between groups

• "U": this code was used where it was difficult to ascertain the direction of the effect (usually due to lack of information).

Individual studies were stratified according to prescribing domain. Within domain we also categorised studies by clinical area. Studies appear more than once if they address decision support across more than one prescribing domain (e.g. initiating and monitoring therapy) or report prescribing outcomes across more than one clinical area. In the latter case, only the prescribing outcomes pertinent to the specific clinical area are presented in each category.

We summarised studies in the manuscript by reporting whether they demonstrated at least one positive prescribing outcome (general trend in favour of CDSS) and statistically significant improvements in favour of CDSS on the majority (≥ 50%) of prescribing outcomes (as used by Garg and colleagues [1]). We chose to report general trends as well as significant results given the likelihood some studies were underpowered to detect statistically significant differences in outcomes. Additional File 2 and Additional File 3 detail results for four summary measures: at least one outcome in favour of CDSS; majority of prescribing outcomes in favour of CDSS; at least one statistically significant prescribing outcome; and statistically significant improvement on the majority of prescribing outcomes.

## Results

### Characteristics of Included Studies (56 studies)

**Figure 1:**
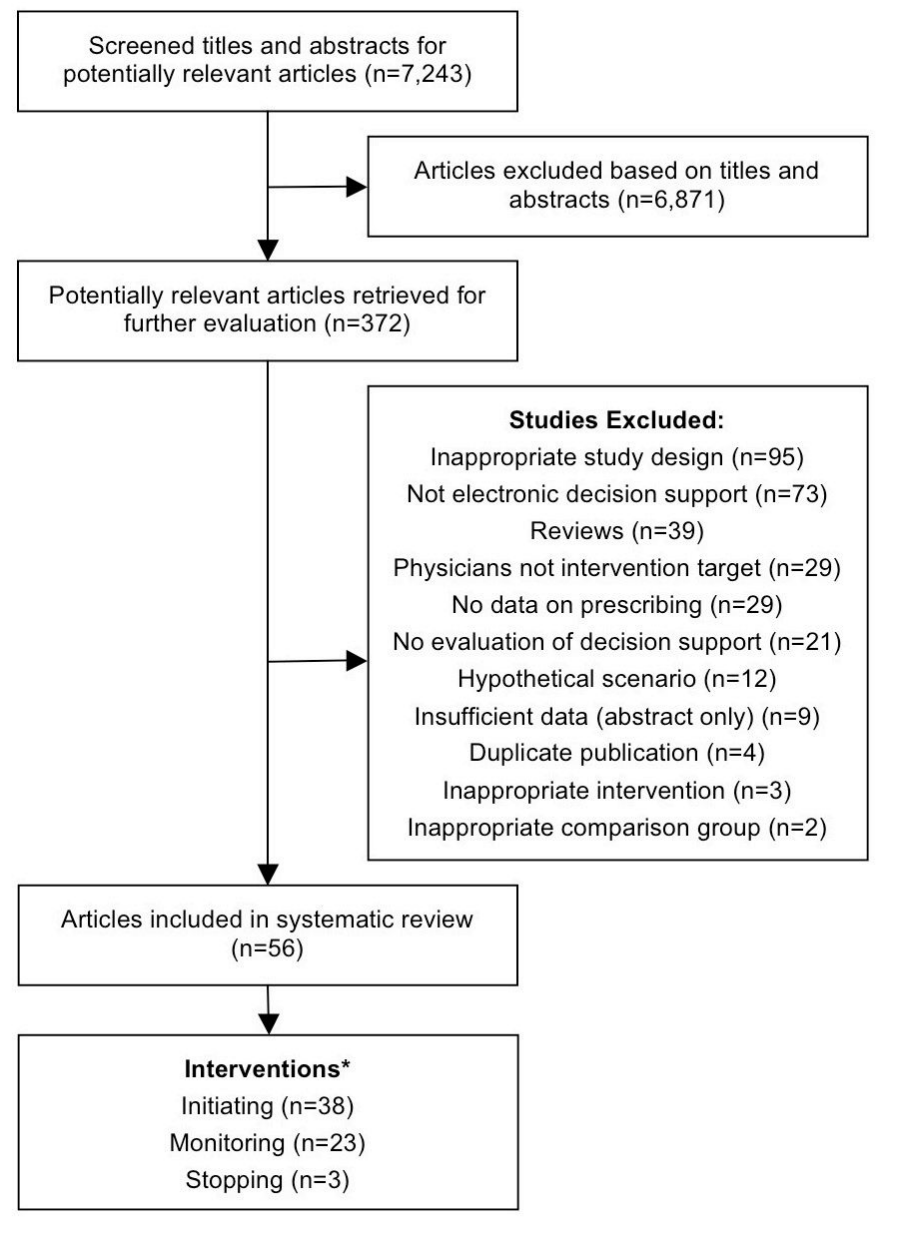
**Process of study inclusion for the systematic review**. * 8 Studies provided data relevant to more than one prescribing domain.

We screened the titles and abstracts of 7,243 articles and reviewed 372 full-text articles. Fifty-six articles were included in the review (Figure 1). Table 2 details the characteristics of these studies.

**Table 2:** Characteristics of included studies

**Characteristic**	**Characteristic Detail**	**Studies (N = 56)**	**References**
Prescribing Domain*	Initiating		
	Before drug selection	26	[16-41]
	After drug selection	12	[42-53]
	Monitoring	23	[20,35,36,39,49,54-71]
	Stopping	3	[39,42,61]

Geographic Setting	North America	39	[16-18,20,21,23,27-30,32,33,35-45,47,49-59,62,65-67]
	Europe	15	[19,22,24-26,31,34,46,48,61,63,64,68-70]
	Other	2	[60,71]

Target	Physicians	35	[19-21,24-27,29-34,37,39,42,44-48,56,57,59-69,71]
	Physicians/other health care professionals	21	[16-18,22,23,28,35,36,38,40,41,43,49-55,58,70]

Initiation of CDSS	System initiated	39	[16,18,20-30,32,33,35-37,40-53,58,60,62,63,66,67,70]
	User initiated	14	[17,31,34,54-57,59,61,64,65,68,69,71]
	Mixed/Unclear	3	[19,38,39]

Clinical Setting	Ambulatory	37	[16-20,22-28,31,32,35-39,41-43,46,48,51-54,58,59,61-64,67-69]
	Institutional	16	[21,29,30,33,34,44,45,47,49,50,55-57,65,66,71]
	Both	3	[40,60,70]

Implementation strategy	CDSS only	37	[17,21,22,27-29,34,36,37,40-57,59-65,68,69,71]
	Multi-faceted	19	[16,18-20,23-26,30-33,35,38,39,58,66,67,70]

Clinical Area*	Cardiovascular disease	19	[16-22,24,26,28,31-33,35,37,38,46,62,67]
	Antibiotic therapy	9	[25,34,44,46,47,50,52,53,56]
	Vaccinations	9	[17,20,21,33,36,38-41]
	Respiratory conditions	9	[22,39,46,48,52,57,64,67,71]
	Anticoagulant therapy	12	[20,21,29,33,54,55,59,63,65,68-70]
	Elderly (multiple conditions and drugs)	4	[42,43,45,49]
	Osteoporosis	2	[23,33]
	Other	11	[20,27,30,36,51,52,58,60,61,66,67]

* Studies are represented more than once across categories if the intervention focused on more than one area

Most studies (n = 38) focussed on decision support at the time of initiating therapy (26 before and 12 after drug selection), 23 on monitoring treatment and three on stopping therapy. Most studies (n = 39) were undertaken in North America, 35 targeted physicians exclusively and 21 targeted physicians with medical students and/or other health care professionals such as nurses, nurse practitioners, pharmacists and physician assistants.

The majority of studies (n = 39) used system-initiated decision support and 37 studies were conducted in ambulatory care (hospital outpatients, Veterans' Medical Centres, Health Maintenance Organisations), 16 in institutional care (hospital inpatient, long-term care) and three across both settings. Nineteen were multi-faceted in that clinicians received combinations of academic detailing, audit and feedback, didactic lectures and written guidelines in addition to decision support.

Cardiovascular disease management was the most common clinical focus (n = 19). Other clinical areas included anticoagulant therapy (n = 12), antibiotic prescribing (n = 9), vaccinations (n = 9), respiratory conditions such as asthma and chronic obstructive pulmonary disease (n = 9), osteoporosis (n = 2) and prescribing for the elderly (n = 4). Fifteen studies addressed more than one clinical area.

### Methodological Quality

Table 3 details the outcomes of the quality assessment. Fifty of the 56 trials were RCTs, six were quasi-experiments, five of which were interrupted time series designs. Fifty studies used random or quasi-random allocation of study groups, 20 reported randomisation by cluster (to minimise contamination) and 24 accounted for clustering in statistical analyses. Thirty six studies reported no baseline differences between study groups or made the appropriate statistical adjustments to account for baseline differences and 53 used objective study outcomes or subjective outcomes with blinded assessment. Fifty trials included at least 90% of baseline participants in final data analyses. On the 10-point methods scale, the mean study quality score was 7.9 (SD 1.9) with a range of 4 to 10.

**Table 3:** Quality assessment

**Item**	**n (%)**
1. Method of allocation of study groups	
2 = Random	48 (86)
1 = Quasi-random	2 (4)
0 = Selected concurrent controls	6 (11)

2. Unit of allocation	
2 = Cluster (e.g. practice)	20 (36)
1 = Physician	13 (23)
0 = Patient	23 (41)

3. Presence of baseline differences between groups	
2 = No baseline differences present or appropriate statistical adjustments made	36 (64)
1 = Baseline differences present and no statistical adjustments made	6 (11)
0 = Baseline characteristics not reported	14 (25)

4. Objectivity of outcome measures	
2 = Objective outcomes or subjective outcomes with blinded assessment	53 (95)
1 = Subjective outcomes with no blinding but clearly defined assessment criteria	3 (5)
0 = Subjective outcomes with no blinding and poorly defined	0 (0)

5. Completeness of follow-up for appropriate unit of analysis	
2 = >90%	50 (89)
1 = 80-90%	2 (4)
0 = <80%	4 (7)

### Impact of CDSSs according to Prescribing Domain

Table 4 summarises the number of studies reporting at least one positive outcome and statistically significant improvement in favour of the CDSS on the majority (≥ 50%) of prescribing outcomes. Additional File 4, Additional File 5, Additional File 6 and Additional File 7 display the key features of individual interventions, outcome measures, quality scores and results, organised by the study's primary clinical focus.

**Table 4:** Studies reporting at least one positive outcome and ≥ 50% significant outcomes in favour of CDSS

	**Before Drug Selection (n = 26) n/N (%)**		**After Drug Selection (n = 12) n/N (%)**		**Monitoring** **(n = 23) n/N (%)**	
	**At least one positive outcome**	**≥ 50% statistically significant outcomes**	**At least one positive outcome**	**≥ 50% statistically significant outcomes**	**At least one positive outcome**	**≥ 50% statistically significant outcomes**

**Overall**	24/26 (92)	12/26 (46)	12/12 (100)	7/12 (58)	18/23 (78)	8/23 (35)

**Initiation of CDSS**						
System	19/20 (95)	12/20 (60)	12/12 (100)	7/12 (58)	9/11 (82)	6/11 (55)
User	2/3 (67)	0/3 (0)	NA	NA	9/11 (82)	2/11 (18)
Mixed/Unclear	3/3 (100)	0/3 (0)	NA	NA	0/1 (0)	0/1 (0)

**Clinical Setting**						
Institutional	5/5 (100)	3/5 (60)	5/5 (100)	4/5 (80)	6/7(86)	3/7 (43)
Ambulatory Care	18/20 (90)	8/20 (40)	7/7 (100)	3/7 (43)	10/14 (71)	4/14 (29)
Both	1/1 (100)	1/1 (100)	NA	NA	2/2 (100)	1/2 (50)

**Mode of Delivery**						
Multi-faceted	13/15 (87)	5/15 (33)	NA	NA	5/7 (71)	2/7 (29)
CDSS only	11/11(100)	7/11 (64)	12/12 (100)	7/12 (58)	13/16 (81)	6/16 (38)

**Clinical Area**						
Cardiovascular	13/16 (81)	4/16 (25)	1/1 (100)	0/1 (0)	2/3 (67)	1/3 (33)
Antibiotics	2/2 (100)	1/2 (50)	6/6 (100)	4/6 (67)	1/1 (100)	0/1 (0)
Vaccinations	8/9 (89)	5/9 (56)	NA	NA	NA	NA
Respiratory	1/2 (50)	0/2 (0)	3/3 (100)	1/3 (33)	2/5 (40)	1/5 (20)
Anticoagulants	3/3 (100)	2/3 (67)	NA	NA	9/9 (100)	2/9 (22)
Elderly	NA	NA	4/4 (100)	2/4 (50)	0/1 (0)	0/1 (0)
Osteoporosis	2/2 (100)	1/2 (50)	NA	NA	NA	NA
Other	3/4 (75)a	3/4 (75)a	2/2 (100)b	2/2 (100)b	5/6 (83)c	4/6 (67)c

a Other clinical areas include: salicylates or paracetamol in patients with history of GI bleed; erythropoietin low Hb; HIV medications; various medications

b Other clinical areas include: various medications interacting with warfarin; various conditions in children (e.g. croup, otitis media)

c Other clinical areas include: hormone treatment for infertility; etretinate for psoriasis; HIV medications; various conditions (e.g. epilepsy, gout, diabetes).

#### Initiating Treatment (38 studies) [16-41]

Overall, 36 studies demonstrated at least one positive prescribing outcome in favour of CDSSs, and 19 reported significant improvements on the majority of outcomes.

#### Before Drug Selection (26 studies)

Almost all studies (n = 24) showed improvements in at least one outcome and 12 demonstrated significant improvements on the majority of outcomes. Importantly, one study involving preventive care recommendations for increased use of angiotensin converting enzyme (ACE) inhibitors [33] found the CDSS was significantly inferior to its comparator on at least one outcome measure.

Nineteen of the 20 system-initiated decision support interventions showed improvements in at least one outcome and 12 demonstrated significant improvements on the majority of outcomes. Two of the three user-initiated systems had a positive effect on at least one outcome but none demonstrated statistically significant improvements.

All but two of the 20 studies conducted in ambulatory care demonstrated at least one positive outcome in favour of CDSS. However, only eight demonstrated significant improvements in the majority of outcomes. All five of the studies conducted in institutional care reported at least one positive outcome in favour of CDSSs, and three demonstrated statistically significant improvements in the majority of measures.

Multi-faceted interventions did not appear to have any advantage over interventions using CDSS alone (with 5 of 15 and 7 of 11 reporting statistically significant results on the majority of outcomes respectively).

Cardiovascular disease was the most common clinical target for CDSS interventions, followed by vaccinations. Sixteen studies addressed aspects of cardiovascular disease management, such as primary and/or secondary prevention. However, only four demonstrated significant positive CDSS impacts on the majority of outcomes measured. All four used system-initiated advice to prompt physicians about the therapeutic management of patients determined to be 'at risk' of cardiovascular events. [21,24,26,28] Five of the nine studies targeting vaccinations demonstrated statistically significant benefits in favour of CDSS for the majority of outcomes [20,21,36,40,41], and they too used system-initiated advice to increase vaccination rates. There were too few studies across the other clinical domains to draw any conclusions about the impact of CDSS in specific clinical areas.

#### After Drug Selection (12 studies) [42-53]

All twelve studies reported improvements in at least one outcome and seven demonstrated favourable results in favour of CDSSs on the majority of outcomes. All of the studies used system-initiated decision support. Four of the five studies conducted in institutional care demonstrated significant improvements in the majority of outcomes and three of the seven studies conducted in ambulatory care demonstrated significant improvements in the majority of outcomes. None of the interventions in this category were multi-faceted.

All six studies examining the effect of CDSS on antibiotic prescribing reported at least one positive outcome, however, four studies demonstrated significant impacts on the majority of outcomes. In each case, the intervention involved system-initiated advice to target the rational use of specific antibiotics.[44,47,50,53] Four studies evaluated the effectiveness of CDSS for prescribing in the elderly. Two demonstrated significant improvements in the majority of outcomes and both studies focussed specifically on safety issues.[43,45] Based on patient specific information, the first intervention customised initial dosing recommendations for sedatives, neuroleptics, anti-emetics and skeletal muscle relaxants. [45] The second used alerts to prompt physicians after prescribing a particular medication to consider alternative therapy. [43]

#### Monitoring Patients on Existing Therapy (23 studies) [20,35,36,39,49,54-71]

Eighteen studies demonstrated positive results on at least one outcome, and eight reported statistically significant results in favour of CDSS on the majority of outcomes. Five studies used CDSSs to advise on maintenance prescribing, such as dose changes, in chronic therapy for drugs including statins, anti-hypertensives, and asthma medications. Twelve studies used dose calculators, eight of which related to warfarin therapy and the remaining studies investigated prescribers' responses to prompts for laboratory tests, such as liver function tests, for a range of medicines; one of these measured adherence to a warfarin monitoring schedule.

Nine of the 11 system-initiated decision support interventions showed improvements in at least one outcome and six demonstrated significant improvements on the majority of outcomes. Nine of the 11 user-initiated systems had a positive effect on at least one outcome but only two demonstrated significant improvements on the majority of outcomes.

Decision support interventions undertaken in institutional care appeared to perform somewhat better than those conducted in ambulatory care (3 of 7 versus 4 of 14 demonstrated the majority of outcomes were statistically significant in favour of CDSS respectively).

We did not find any differences in the effectiveness of multifaceted interventions versus CDSS only (with 2 of 7 and 6 of 16 reporting statistically significant results on the majority of outcomes respectively).

All nine studies involving warfarin (anticoagulant) therapy reported at least one positive outcome. Only two of these demonstrated significant improvements in the majority of outcomes. There were too few studies across the other clinical domains to draw any conclusions about the impact of CDSS in specific clinical areas. However, there was some evidence to support CDSS in the realm of reminders for laboratory tests with four of the seven studies reporting statistically significant results favouring CDSS on the majority of outcomes. All of the effective studies used system-initiated advice to increase therapeutic monitoring of a range of drugs. [36,58,60,66]

#### Stopping Treatment (3 studies) [39,42,61]

Studies in this domain were too few to draw any specific conclusions relating to our hypotheses. Two studies were conducted in institutional care. One addressed stopping ipratropium therapy and found the intervention inferior to standard care (although the result was not statistically significant).[39] The second hospital-based study involved patients undergoing ovarian stimulation for infertility using software to predict whether additional cycles of ovulatory stimulants should be administered [61] however the impact on the prescribing outcome (number of cancelled cycles) was unclear. There were no statistically significant changes in any of the prescribing outcomes for the third study that examined alerts relating to discontinuing potentially inappropriate medications in the elderly (e.g. NSAIDs and benzodiazepines). [42]

## Discussion

The current review explored the impact of CDSSs on key decision points in the prescribing process and highlights the diversity of studies, intervention targets and methods reported to date. While the range and variety of studies adds great breadth to the field it makes synthesis challenging and creates difficulties in providing clear guidance on where CDSSs are likely to be most effective. We did however find some general trends supporting the effectiveness of CDSSs across specific aspects of the prescribing process.

### Key Findings

In the realm of initiating therapy we found some indication of greater effectiveness of CDSSs after, rather than before, drug selection. The effective interventions implemented after drug selection had taken place all used system-initiated advice to flag key safety issues (such as alerting providers to high severity drug interactions, contraindications with other medications and cautions against prescribing particular medications for the elderly) or provided quality use of medicine messages (such as alterations to durations of therapy and/or form of prescribed drugs). This supports our hypothesis that it is easier to influence practice in relation to fine-tuning therapy rather than attempting to influence initial therapeutic choices. We also found some evidence to support the benefits of CDSSs in increasing the laboratory testing rates for patients on long-term therapy including cardiovascular and respiratory medicines. Further, consistent with previous reviews, system-initiated CDSSs appeared to be more effective than user-initiated systems as did CDSSs implemented in institutional as opposed to ambulatory settings.

We did not however find any evidence to support the notion that CDSSs implemented with other strategies such as education are more successful in changing prescribing than stand alone interventions. It is notable that few studies addressed stopping or tapering therapy, none of which demonstrated impacts in favour of CDSSs over comparators on the majority of outcomes. While many of the interventions addressed the prevention and management of cardiovascular disease only a small number of these demonstrated significant improvements on the majority of prescribing outcomes. Studies were too few in the other clinical domains to draw firm conclusions about the benefits of CDSS in managing specific clinical areas.

### Limitations

This review had a number of limitations. Despite our intensive efforts, the collection of intervention studies is likely to be incomplete as some evaluations may not be available in the public domain, others may be published outside the peer-reviewed academic literature or published in languages other than English. Of the available studies, it was difficult to synthesise the material presented due to use of ambiguous terminology and more particularly there was little detail provided about the way in which the CDSSs were integrated into existing and/or new software systems. We did however manage to extract some key information about system and organisational factors demonstrated in previous reviews to influence outcomes.

While our review is not unique to the field, the overlap between studies in our review and others published previously is surprisingly small (see Additional File 8). This is due primarily to the variability in search terms and dates and more specifically inclusion and exclusion criteria (for example, RCTs versus RCTs plus quasi-experiments; physicians only versus all providers; prescribing only versus the full spectrum of clinical care; computerised advice delivered in electronic formats versus other electronic and/or paper-based formats). Prescribing is a key aspect of modern medicine therefore there is a strong case to add another review to the existing evidence by examining where CDSSs have the greatest impact across the prescribing process. Importantly, as the evidence base grows and reporting of CDSS and organisational features improves, a clear enhancement to the current review would be assessment of the impact of additional system and organisational features on user acceptability and likelihood of changes in practice in line with CDSS recommendations.

Previous reviews in prescribing have been more restricted than ours and even the most recent and extensive of these reviews [9] only targets initiation of therapy and ignores other prescribing dimensions associated with reviewing/monitoring existing therapies and stopping therapies. CDSS interventions are highly complex and their effectiveness is dependent largely on how well they are designed and implemented. This review was not able to consider the full impact of factors such as system design, usability and integration with clinician workflow which may have influenced the ability of systems to deliver anticipated prescribing outcomes.

There are also fundamental difficulties in comparing interventions with diverse objectives, measurement methods and outcomes. We managed this issue by reporting differences in favour of CDSSs on at least one prescribing outcome and significant differences on the majority of prescribing outcomes. Many results were positive, but not statistically significant, suggesting inadequate sample sizes and under powering of studies. There is also the possibility that there is some bias in publishing 'positive' studies, however many studies included in this review did not demonstrate significant outcomes in favour of CDSS. We replicated the summary measure used by Garg et al [1] (≥ 50% of significant outcomes), but it may be influenced by the number of outcomes reported and the appropriateness of the outcomes used. For example, the more prescribing outcomes reported, the more difficult it may be to achieve significant positive effects on the majority of outcomes measured. However, had we based our findings on the outcome 'at least one statistically significant outcome in favour of CDSS', our conclusions would change only for setting. Using this less stringent measure, we would have concluded there was no evidence indicating that CDSS was more effective in institutional compared with ambulatory care settings.

Many of the studies in this review used outcomes such as prescribing volume or rates (i.e. number of prescriptions for a particular drug or drug class). While these measures are clearly indicative of a desired change in prescribing practice, previous research has demonstrated that changes in these measures do not always equate to more 'appropriate' prescribing and better outcomes for all patient subgroups. [72,73] Thus, the choice of outcome measures sensitive to such issues would be of greater benefit in establishing intended and unintended consequences of any intervention.

While relatively few studies achieved significant positive effects on the majority of outcomes, considerably more studies did demonstrate at least one positive intervention effect. Many of the studies in this review addressed common conditions and drugs so any change in outcome may equate to substantial clinical and fiscal benefits. Further, while this review did not specifically address the impact of CDSSs on patient outcomes, if the change in a prescribing outcome has been demonstrated in randomised controlled trials to translate into clinical benefit for specific patient populations (e.g. prescribing medicines to lower blood pressure or to lower cholesterol levels leads to fewer myocardial infarctions and strokes) this adds further weight to the benefits of these interventions.

Further, 'neutral' studies should not necessarily be viewed negatively. While it was not the intent of the interventions in this review to establish 'equivalence' it may be worth considering the benefits of CDSSs in releasing health care professionals from activities they need not undertake, such as actively documenting when tests and follow-up should be performed. Finally, few studies reported unintended negative consequences of CDSS interventions such as undesirable drug substitution effects. While our review did not raise any particular concerns about the negative effect of CDSS, its full impact remains unknown.

### Commentary

Limitations notwithstanding, this study has contributed to the literature by detailing the specific areas within the prescribing process where CDSSs tend to be more effective. The most consistently effective approaches used system-initiated decision support to fine-tune existing therapy by making recommendations to improve patient safety, adjust the dose, duration or form of prescribed drugs or increase the laboratory testing rates for patients on long-term therapy. This finding remains consistent with other literature. Patient safety is a predominant feature of clinical culture and any system highlighting potential problems is welcomed and adopted by clinicians. [1,7] These triggers and reminders simply add safeguards and enhance continuity and long-term care rather than posing any threat to professional autonomy.

There is currently insufficient evidence to draw conclusions about the impact of CDSSs with respect to stopping therapy but changing behaviour in this domain will likely bring its own challenges. Prescribers may be unwilling to cease drugs that appear to be working and importantly doing no harm; and patients are unlikely to request the drug be stopped. The result is that therapy once started may be continued long term. Further, reminders for this kind of clinical review may be perceived as undermining professional autonomy. [74] Clearly, more studies need to be conducted in this domain to determine if these issues apply.

Our findings that system-initiated decision support systems are more effective than user-initiated systems, and institutional interventions are more effective than those conducted in community practice, are not surprising. Compared with decision support where users are required to initiate systems manually, automatic prompts are likely to dovetail more closely with physician workflow and provide opportunities to work on fine-tuning decisions or correct shortfalls or errors in care.[1,2] The success of institutional interventions is likely attributed to the type of conditions managed in this setting, the stricter controls on the practices of health care professionals in institutional settings and a potentially greater willingness to abide by 'externally' imposed rules and management suggestions.

Our finding that multi-faceted interventions appeared no more effective than CDSS interventions alone conflicts with a number of other reviews [12-14] and also appears counterintuitive from a behavioural perspective. However, in a review of more than 200 studies assessing the effectiveness of guideline implementation strategies, Grimshaw and colleagues [75] found multi-faceted interventions did not appear to be more effective than single strategy interventions but emphasised the difficultly in drawing generalisable conclusions about multi-faceted interventions owing to the large number of different combinations used. Further, our results are likely to be confounded by the setting in which the interventions occurred, clinical condition being targeted and the system design and implementation.

## Conclusion

With the ever increasing enthusiasm for, and implementation of CDSSs in medical practice it is paramount we determine their benefits and risks in the short, medium and longer term. As such, it is important to promote the rigorous testing of these systems to produce high quality evidence about their clinical and economic impacts. Our review and others demonstrate CDSSs have merit in supporting specific aspects of the prescribing process. However, despite the substantial number of CDSS interventions in this review our understanding of the impacts of these interventions in particular clinical domains and settings is still relatively limited. Further, interventions to date have largely focussed on supporting clinicians in initiating and monitoring rather than ceasing therapy.

System developers and policy makers should build on the consistently effective CDSS approaches identified in this research such as prompts and alerts incorporating 'do no harm messages', reminders for patients on long-term therapy and care suggestions for 'at risk' patients. In addition, further studies are required to determine the relative merits of particular types of CDSS and the factors related to the successful implementation of these systems. Our understanding of the impact of CDSSs in clinical practice would be much enhanced by detailed reporting on the individual features of the systems, the way in which they were developed and the specifics of the environment in which they were deployed.

## Competing interests

The authors declare that they have no competing interests.

## Authors' contributions

SP conceived and designed the study, participated in data collection and analysis, interpreted findings and drafted the manuscript. AM contributed to the study design, data collection and analysis and interpretation of findings. JR conceived and designed the study and participated in data collection and analysis and interpretation of findings. IH contributed to the study design, data collection and analysis and interpretation of findings. MW contributed to the study design and interpretation of findings. JR contributed to the study design and interpretation of findings. DN conceived and designed the study and participated in data collection and analysis and interpretation of findings. All authors read and approved the final manuscript.

## Pre-publication history

The pre-publication history for this paper can be accessed here:



## Supplementary Material

Additional file 1 **Full search strategy.** Click here for file

Additional file 2 **Table S1 - Full summary of results - Initiating treatment**. a Other clinical areas include: salicylates or paracetamol in patients with history of GI bleed; erythropoietin low Hb; HIV medications; various medications. b Other clinical areas include: various medications interacting with warfarin; various conditions in children (e.g. croup, otitis media) Click here for file

Additional file 3 **Table S2 - Full summary of results - Monitoring treatment**. c Other clinical areas include: hormone treatment for infertility; etretinate for psoriasis; HIV medications; various conditions (e.g. epilepsy, gout, diabetes). Click here for file

Additional file 4 **Table S3: Key study features and results (Initiating treatment - Before drug selection)**. * Unless otherwise stated, number of patients is close to or equal to that specified in the "participants" column, or was not reported. + (NS) indicates intervention favoured the CDSS but was not statistically significant; - (NS) indicates intervention favoured comparison group but was not statistically significant; 0 = no difference between groups; **++ **indicates intervention favoured CDSS and was statistically significant; **- - **indicates intervention favoured comparator and was statistically significant; U = unclear. ACE = angiotensin-converting enzyme; BMD = bone mineral density; BP = blood pressure; CAD = coronary artery disease; CDSS = computerised clinical decision support system; CHD = coronary heart disease; CHF = congestive heart failure; CME = continuing medical education; CPOE = computerised provider order entry; COPD = chronic obstructive pulmonary disease; CVD = cardiovascular disease; EMR = electronic medical record; GI = gastro intestinal; GP = general practitioner; Hgb = haemoglobin; HIV = human immuno-deficiency virus; HMO = Health Maintenance Organisation; IHD = ischemic heart disease; LDL = low-density lipoprotein; MI = myocardial infarction; NSAIDs = non-steroidal anti-inflammatory drugs; NYHA = New York Heart Association; PCP = P carinii pneumonia; RCT = randomised controlled trial; UTI = urinary tract infection; Click here for file

Additional file 5 **Table S4: Key study features and results (Initiating treatment - After drug selection)**. * Unless otherwise stated, number of patients is close to or equal to that specified in the "participants" column, or was not reported. + (NS) indicates intervention favoured the CDSS but was not statistically significant; - (NS) indicates intervention favoured comparison group but was not statistically significant; 0 = no difference between groups; **++ **indicates intervention favoured CDSS and was statistically significant; **- - **indicates intervention favoured comparator and was statistically significant; U = unclear. CDSS = computerised clinical decision support system; CPOE = computerised provider order entry; COPD = chronic obstructive pulmonary disease; CNS = central nervous system; CVD = cardiovascular disease; ED = emergency department; EMR = electronic medical record; GP = general practitioner; HMO = Health Maintenance Organisation; ICU = intensive care unit; IV = intravenous; MRSA = methicillin-resistant *Staphylococcus aureus*; NSAIDs = non-steroidal anti-inflammatory drugs; RCT = randomised controlled trial; TCA = tertiary amine tricyclics antidepressant. Click here for file

Additional file 6 **Table S5: Key study features and results (Monitoring existing therapy)**. * Unless otherwise stated, number of patients is close to or equal to that specified in the "participants" column, or was not reported. + (NS) indicates intervention favoured the CDSS but was not statistically significant; - (NS) indicates intervention favoured comparison group but was not statistically significant; 0 = no difference between groups; **++ **indicates intervention favoured CDSS and was statistically significant; **- - **indicates intervention favoured comparator and was statistically significant; U = unclear. ACE = angiotensin-converting enzyme; BP = blood pressure; ALT = alanine aminotransferase; AST = aspartate aminotransferase; CBC = complete blood count; CDSS = computerised clinical decision support system; CHD = coronary heart disease; CME = continuing medical education; CPOE = computerised provider order entry; COPD = chronic obstructive pulmonary disease; EMR = electronic medical record; FSH = follicle-stimulating hormone; HIV = human immuno-deficiency virus; HMO = health maintenance organisation; INR = international normalised ratio; IV = intravenous; LFT = liver function test; LH = luteinizing hormone; NYHA = New York Heart Association; PTR = prothrombin time ratio; RCT = randomised controlled trial; TSH = thyroid stimulating hormone; Click here for file

Additional file 7 **Table S6: Key study features and results (Stopping treatment)**. * Unless otherwise stated, number of patients is close to or equal to that specified in the "participants" column, or was not reported. + (NS) indicates intervention favoured the CDSS but was not statistically significant; - (NS) indicates intervention favoured comparison group but was not statistically significant; 0 = no difference between groups; **++ **indicates intervention favoured CDSS and was statistically significant; **- - **indicates intervention favoured comparator and was statistically significant; U = unclear. CDSS = computerised clinical decision support system; CPOE = computerised provider order entry; COPD = chronic obstructive pulmonary disease; EMR = electronic medical record; FSH = follicle-stimulating hormone; GP = general practitioner; LH = luteinizing hormone; NSAID = non-steroidal anti-inflammatory drugs; NYHA = New York Heart Association; RCT = randomised controlled trial. Click here for file

Additional file 8 **Table S7: Key differences between the studies included in the current review and previous reviews**. * Studies were not prescribing focused (screening, preventive care/disease management). † Studies addressed automated drug dosing studies without decision support. # Review excluded studies below a defined quality rating. ‡ Review did not include monitoring or ceasing studies. Click here for file
