# Supplementary material for: Do computerised clinical decision support systems for prescribing change practice? A systematic review of the literature (1990-2007)
Source: BMC Health Serv Res. 2009 Aug 28;9:154. doi: 10.1186/1472-6963-9-154 (PMC2744674; doi:10.1186/1472-6963-9-154)
Supplement: Additional file 1 — Full search strategy. [file 1472-6963-9-154-S1.doc]

**Full search strategy**

**Combined Search**

Medline (1966 – November Week 3, 2006); Embase (1980 – Week 47, 2006); Cinahl (1982 – November Week 4, 2006); PsycINFO (1985 – November Week 4, 2006)

**# Search History Results**

1 decision support systems, clinical/ 3356

2 decision support system/ 1776

3 exp decision support techniques/ 46337

4 exp decision making, computer-assisted/ 43010

5 computer assisted drug therapy/ 1065

6 computer assisted diagnosis/ 19781

7 computer-assisted instruction/ 24545

8 medical informatics/ 7302

9 medical informatics computing/ 3450

10 medical informatics applications/ 4166

11 expert systems/ 6825

12 ((computer$ or electronic$) adj5 (guideline$ or 9519

protocol$ or alert$ or reminder$ or audit$ or

feedback$)).mp.

13 ((computer$ or electronic$) adj5 decision$).mp. 7867

14 or/1-13 144117

15 decision support$.mp. 16010

16 decision making/ 98046

17 medical decision making/ 41129

18 clinical decision making/ 6511

19 evidence-based medicine/ 57044

20 practice guidelines/ 126082

21 guideline adherence/ 86760

22 clinical protocols/ 47567

23 reminder systems/ 1588

24 education, medical/ 90123

25 education, medical, continuing/ 71708

26 education, continuing/ 21960

27 adverse drug reaction reporting systems/ 8492

28 drug information services/ 15104

29 drug information/ 12108

30 clinical pharmacy information systems/ 6435

31 medical information/ 27327

32 or/15-31 522649

33 exp computer systems/ 180425

34 online systems/ 10345

35 internet/ 56392

36 user-computer interface/ 15809

37 (computer$ or electronic$ or online or internet).mp. 955330

38 or/33-37 1018383

39 32 and 38 50889

40 14 or 39 177692

41 prescriptions, drug/ 58042

42 (prescribing or prescription$).mp. 128264

43 drug utilization/ 18572

44 drug interactions/ 111527

45 drug monitoring/ 34636

46 drug therapy, combination/ 109393

47 drug therapy.mp. 411470

48 drug choice/ 11526

49 drug substitution/ 1577

50 drug contraindication/ 11653

51 drug dose regimen/ 17987

52 drug delivery systems/ 44993

53 exp pharmaceutical preparations/ 1135856

54 pharmaceutical care/ 7658

55 medication$.mp. 257656

56 or/41-55 1993923

57 40 and 56 10913

58 limit 57 to English language 9863

59 limit 58 to humans [Limit not valid in: CINAHL 8702

PsycINFO; records were retained]

60 limit 59 to yr=”1990-2006” 8338

61 remove duplicates from 60 7427

62 (letter or editorial).pt. 1398380

63 60 not 62 **6933** **(Total)**

mp = ti, hw, ab, it, sh, tn, ot, dm, mf, nm, tc, id

= title, subject heading word, abstract, instrumentation, subject heading, drug trade name, original title, device manufacturer, drug manufacturer name, name of substance word, table of contents, identifiers

**Pre-Medline (30 November, 2006)**

[Note: above search was re-run; only terms that returned citations are listed]

**# Search History Results**

1 ((computer$ or electronic$) adj5 (guideline$ or 105

protocol$ or alert$ or reminder$ or audit$ or

feedback$)).mp.

2 ((computer$ or electronic$) adj5 decision$).mp. 50

3 or/1-2 149

4 decision support$.mp. 198

5 decision making/ 3

6 or/4-5 201

7 (computer$ or electronic$ or online or internet).mp. 16646

8 6 and 7 84

9 3 or 8 191

10 (prescribing or prescription$).mp. 1589

11 drug therapy.mp. 465

12 medication$.mp. 3763

13 or/10-12 5362

14 9 and 13 27

15 limit 14 to English language 27

16 limit 15 to humans [Limit not valid; records 27

were retained]

17 (physician$ or clinician$ or practitioner$ or 8746

doctor$).mp.

18 (GPs or GP).mp. 836

19 general pract$.mp. 892

20 (provider or hospital).mp. 10058

21 or/17-20 18374

22 16 and 21 23

23 (letter or editorial).pt. 16149

24 22 not 23 **23 (first scan)**

25 16 not 22 4

26 25 not 23 **4 (second scan)**

27 24 or 26 **27 (total)**

**Cochrane Database of Systematic Reviews (November 2006) Search**

**# Search History Results**

1 EPOC list of protocols and reviews 71

2 (decision support):ti,ab,kw 42

**INSPEC (1969 – November 2006)**

**# Search History Results**

1 TX decision support 24189

2 TX prescribing 926

3 TX medication 921

4 TX drug 13027

5 TX prescription 7030

6 (S5 or S4 or S3 or S2) 21179

7 (S6 and S1) 267

8 Limiters – Date: 1990-200611 234

| **Database** | **Number of Unique Citations** |
| --- | --- |
| Medline | 3791 |
| Embase | 2571 |
| Cinahl | 501 |
| PsycINFO | 70 |
| Pre-Medline | 19 |
| INSPEC | 181 |
| Cochrane Database | 110 |
| **Total** | **7243** |
