# Supplementary material for: Do computerised clinical decision support systems for prescribing change practice? A systematic review of the literature (1990-2007)
Source: BMC Health Serv Res. 2009 Aug 28;9:154. doi: 10.1186/1472-6963-9-154 (PMC2744674; doi:10.1186/1472-6963-9-154)
Supplement: Additional file 2 — Table S1 - Full summary of results - Initiating treatment. a Other clinical areas include: salicylates or paracetamol in patients with history of GI bleed; erythropoietin low Hb; HIV medications; various medications. b Other clinical areas include: various medications interacting with warfarin; various conditions in children (e.g. croup, otitis media) [file 1472-6963-9-154-S2.doc]

**Table Ia – Full summary of results – Initiating treatment**

|  | **Before Drug Selection (n=26) n/N (%)** | | | | **After Drug Selection (n=12) n/N (%)** | | | |
| --- | --- | --- | --- | --- | --- | --- | --- | --- |
|  | **At least one positive outcome** | **>50% positive outcomes** | **At least one statistically significant outcome** | **>50% statistically significant outcomes** | **At least one positive outcome** | **>50% positive outcomes** | **At least one statistically significant outcome** | **>50% statistically significant outcomes** |
| **Overall** | 24/26 (92) | 21/26 (81) | 15/26 (58) | 12/26 (46) | 12/12 (100) | 11/12(92) | 12/12 (100) | 7/12 (58) |
| **Initiation of CDSS** |  |  |  |  |  |  |  |  |
| System | 19/20 (95) | 17/20 (85) | 14/20 (70) | 12/20 (60) | 12/12 (100) | 11/12 (92) | 12/12 (100) | 7/12 (58) |
| User | 2/3 (67) | 3 (67) | 0/3 (0) | 0/3 (0) | NA | NA | NA | NA |
| Mixed / Unclear | 3/3 (100) | 2/3 (67) | 1/3 (33) | 0/3 (0) | NA | NA | NA | NA |
| **Clinical Setting** |  |  |  |  |  |  |  |  |
| Institutional | 5/5 (100) | 5/5 (100) | 3/5 (60) | 3/5 (60) | 5/5 (100) | 5/5 (100) | 5/5 (100) | 4/5 (80) |
| Ambulatory Care | 18/20 (90) | 15/20 (75) | 11/20 (55) | 8/20 (40) | 7/7 (100) | 6/7 (86) | 7/7 (100) | 3/7 (43) |
| Both | 1/1 (100) | 1/1 (100) | 1/1 (100) | 1/1 (100) | NA | NA | NA | NA |
| **Mode of Delivery** |  |  |  |  |  |  |  |  |
| Multi-faceted | 13/15 (87) | 11/15 (73) | 7/15 (47) | 5/15 (33) | NA | NA | NA | NA |
| CDSS alone | 11/11(100) | 10/11 (91) | 8/11 (73) | 7/11 (64) | 12/12 (100) | 11/12 (92) | 12/12 (100) | 7/12 (58) |
| **Clinical Area** |  |  |  |  |  |  |  |  |
| Cardiovascular | 13/16 (81) | 9/16 (56) | 6/16 (38) | 4/16 (25) | 1/1 (100) | 1/1 (100) | 0/1 (0) | 0/1 (0) |
| Antibiotics | 2/2 (100) | 2/2 (100) | 1/2 (50) | 1/2 (50) | 6/6 (100) | 6/6 (100) | 6/6 (100) | 4/6 (67) |
| Vaccinations | 8/9 (89) | 8/9 (89) | 5/9 (56) | 5/9 (56) | NA | NA | NA | NA |
| Respiratory | 1/2 (50) | 1/2 (50) | 0/2 (0) | 0/2 (0) | 3/3 (100) | 2/3 (67) | 3/3 (100) | 1/3 (33) |
| Anticoagulants | 3/3 (100) | 3/3 (100) | 2/3 (67) | 2/3 (67) | NA | NA | NA | NA |
| Elderly | NA | NA | NA | NA | 4/4 (100) | 4/4 (100) | 4/4 (100) | 2/4 (50) |
| Osteoporosis | 2/2 (100) | 2/2 (100) | 1/2 (50) | 1/2 (50) | NA | NA | NA | NA |
| Other | 3/4 (75)a | 3/4 (75)a | 3/4 (75)a | 3/4 (75)a | 2/2 (100)b | 2/2 (100)b | 2/2 (100)b | 2/2 (100)b |

a Other clinical areas include: salicylates or paracetamol in patients with history of GI bleed; erythropoietin low Hb; HIV medications; various medications. b Other clinical areas include: various medications interacting with warfarin; various conditions in children (e.g. croup, otitis media)
