# Supplementary material for: Do computerised clinical decision support systems for prescribing change practice? A systematic review of the literature (1990-2007)
Source: BMC Health Serv Res. 2009 Aug 28;9:154. doi: 10.1186/1472-6963-9-154 (PMC2744674; doi:10.1186/1472-6963-9-154)
Supplement: Additional file 3 — Table S2 - Full summary of results - Monitoring treatment. c Other clinical areas include: hormone treatment for infertility; etretinate for psoriasis; HIV medications; various conditions (e.g. epilepsy, gout, diabetes). [file 1472-6963-9-154-S3.doc]

**Table Ib – Full summary of results – Monitoring treatment**

|  | **Monitoring (n=23) n/N (%)** | | | |
| --- | --- | --- | --- | --- |
|  | **At least one positive outcome** | **>50% positive outcomes** | **Any statistically significant outcome** | **>50% statistically significant outcomes** |
| Overall | 18/23 (78) | 16/23 (70) | 15/23 (65) | 8/23 (35) |
| **Initiation of CDSS** |  |  |  |  |
| System | 9/11 (82) | 9/11 (82) | 8/11 (73) | 6/11 (55) |
| User | 9/11 (82) | 8/11 (73) | 7/11 (64) | 2/11 (18) |
| Mixed / Unclear | 0/1 (0) | 0/1 (0) | 0/1 (0) | 0/1 (0) |
| **Clinical Setting** |  |  |  |  |
| Institutional | 6/7(86) | 5/7 (71) | 4/7 (57) | 3/7 (43) |
| Ambulatory Care | 10/14 (71) | 9/14 (64) | 9/14 (64) | 4/14 (29) |
| Both | 2/2 (100) | 2/2 (100) | 2/2 (100) | 1/2 (50) |
| **Mode of Delivery** |  |  |  |  |
| Multi-faceted | 5/7 (71) | 4/7 (57) | 4/7 (57) | 2/7 (29) |
| CDSS only | 13/16 (81) | 12/16 (75) | 11/16 (69) | 6/16 (38) |
| **Clinical Area** |  |  |  |  |
| Cardiovascular | 2/3 (67) | 2/3 (67) | 2/3 (67) | 1/3 (33) |
| Antibiotics | 1/1 (100) | 1/1 (100) | 1/1 (100) | 0/1 (0) |
| Vaccinations | NA | NA | NA | NA |
| Respiratory | 2/5 (40) | 1/5 (20) | 1/5 (20) | 1/5 (20) |
| Anticoagulants | 9/9 (100) | 9/9 (100) | 7/9 (78) | 2/9 (22) |
| Elderly | 0/1 (0) | 0/1 (0) | 0/1 (0) | 0/1 (0) |
| Osteoporosis | NA | NA | NA | NA |
| Other | 5/6 (83)c | 4/6 (67)c | 5/6 (83)c | 4/6 (67)c |

c Other clinical areas include: hormone treatment for infertility; etretinate for psoriasis; HIV medications; various conditions (e.g. epilepsy, gout, diabetes).
