# Supplementary material for: Do computerised clinical decision support systems for prescribing change practice? A systematic review of the literature (1990-2007)
Source: BMC Health Serv Res. 2009 Aug 28;9:154. doi: 10.1186/1472-6963-9-154 (PMC2744674; doi:10.1186/1472-6963-9-154)
Supplement: Additional file 4 — Table S3: Key study features and results (Initiating treatment - Before drug selection). * Unless otherwise stated, number of patients is close to or equal to that specified in the "participants" column, or was not reported. + (NS) indicates intervention favoured the CDSS but was not statistically significant; - (NS) indicates intervention favoured comparison group but was not statistically significant; 0 = no difference between groups; ++ indicates intervention favoured CDSS and was statistically significant; - - indicates intervention favoured comparator and was statistically significant; U = unclear. ACE = angiotensin-converting enzyme; BMD = bone mineral density; BP = blood pressure; CAD = coronary artery disease; CDSS = computerised clinical decision support system; CHD = coronary heart disease; CHF = congestive heart failure; CME = continuing medical education; CPOE = computerised provider order entry; COPD = chronic obstructive pulmonary disease; CVD = cardiovascular disease; EMR = electronic medical record; GI = gastro intestinal; GP = general practitioner; Hgb = haemoglobin; HIV = human immuno-deficiency virus; HMO = Health Maintenance Organisation; IHD = ischemic heart disease; LDL = low-density lipoprotein; MI = myocardial infarction; NSAIDs = non-steroidal anti-inflammatory drugs; NYHA = New York Heart Association; PCP = P carinii pneumonia; RCT = randomised controlled trial; UTI = urinary tract infection; [file 1472-6963-9-154-S4.doc]

**Table II**: Key study features and results (Initiating treatment – Before drug selection)

| Study | **Setting** | Participants | **Intervention** | **Comparison** | **QA Score (N/10)** | **Practice change in line with intent of CDSS *** | **Change in performance** |
| --- | --- | --- | --- | --- | --- | --- | --- |
| **Cardiovascular** | | | | | | | |
| Ansari  2003[41]  US  RCT | Veterans Affairs medical centre  *Ambulatory care* | Provider – General internists, cardiologists, internal medicine, residents, nurse practitioners (n=74)  Patients – CHF, left ventricular ejection fraction ≤45%, no contraindications to β-blockers, not receiving target dose (n=169) | Alert flagging patients eligible for treatment.  Plus printed and online guidelines, didactic group lectures and patient letters to discuss β-blocker treatment with provider.  *System initiated*  *Multi-faceted intervention* | Usual care  Plus written and online guidelines and didactic group lectures | 9 | Start β-blockers (n=115) | **–** (NS) |
|  |  |  |  |  |  | Start or ↑ β-blockers (n=115) | **–** (NS) |
| Apkon  2005 [42]  US  RCT | Military health practices (n=2)  *Ambulatory care* | Provider – Physicians (n=8), nurse practitioner (n=1), physician assistants (n=3)  Patients – Age>18 years, English speaking with no emergency or obstetric conditions (n=1,902) | Care suggestions after input of medical history and screening information by patients and providers. Information linked to propriety medical database and output detailed diagnosis and treatment options.  *User initiated*  *CDSS only* | Usual care | 8 | Start ACE inhibitors for diabetics (n=3) | – (NS) |
| Bloomfield  2005 [43]  US  RCT | Veterans Affairs medical centres (n=5)  *Ambulatory care* | Providers – Physicians, nurse practitioners, physician assistants (n=92)  Patients – Diagnosis of IHD, high cholesterol, not receiving lipid lowering medication (n=1,349) | 1. Reminder about lipid management in patient EMR at time of visit.  2. Reminder about lipid management and when logged onto patient record system were notified to co-sign progress notes of specific patients.  Plus written education materials, didactic lecture, opinion leaders.  *System initiated*  *Multi-faceted intervention* | Patient letters  Plus written education materials, didactic lecture, opinion leaders | 7 | ↑ Lipid medications (fibrates, statins, bile acid binding resins, niacin) | 1) + (NS)  2) + (NS) |
| Cobos  2005 [44]  Spain  RCT | General practices (n=42)  *Ambulatory care* | Provider – GPs  Patients – High cholesterol, currently on medication or new to treatment (n=2,191) | Care suggestions on therapy, follow-up visits and laboratory tests.  Plus patient health promotion.  *Unclear*  *Multi-faceted intervention* | Usual care | 9 | ↓ Lipid lowering drugs in low risk patients with no CHD | **++** |
|  |  |  |  |  |  | *↑ Use of preferred alternative lipid lowering drugs:*  *↑* Simvastatin | + (NS) |
|  |  |  |  |  |  | ↑ Fluvastatin | + (NS) |
|  |  |  |  |  |  | Appropriate use of lipid lowering drugs in patients with CHD | U |
|  |  |  |  |  |  | Appropriate use of lipid lowering drugs in high risk patients with no CHD | U |
| Demakis  2000 [21]  US  RCT | Veterans Affairs hospital outpatient centres (n=12)  *Ambulatory care* | Provider – Resident physicians (n=275)  Patients – Eligible for 1 or more standards of care (n=12,989) | Reminder about patients who were suitable for “standards of care review”. Rationale for standard also provided with reminder.  Paper version also provided plus didactic group lecture, written materials.  *System initiated*  *Multi-faceted intervention* | Usual care  Plus didactic group lecture, written materials | 9 | Use of β-blockers <1 year after MI (n=609) | + (NS) |
|  |  |  |  |  |  | Use of warfarin, aspirin or ticlopidine for atrial fibrillation (n=477) | – (NS) |
|  |  |  |  |  |  | Switch to salicylates or paracetamol for patients with history of GI bleed taking NSAIDs (n=964) | – (NS) |
| Dexter  2001 [17]  US  RCT | Hospital inpatient (n=8 teams)  *Institutional care* | Provider – General medicine residents and medical students (n=202)  Patients – All admitted to general medicine service (n=10,065 admissions for 6,371 patients) | Rule-based reminders generated when the patient’s EMR included at least 1 indication for the selected preventive therapies.  Plus usual CPOE.  *System initiated*  *CDSS only* | Usual care (CPOE) | 10 | ↑ Aspirin at discharge (n=1,698) | **++** |
| Eccles  2002 [45]  UK  RCT | General practices (n=60)  *Ambulatory care* | Provider – GPs (4.5 partners per practice) and practice nurses. Single-handed practices excluded.  Patients – Age>18 years with angina (n=2,881 with prescribing data) | Information in patients’ EMR triggered guideline and presentation of patient scenarios on angina. System offered management suggestions and requested entry of relevant information to be stored in EMR.  *System initiated*  *CDSS only* | Usual care (computerised guideline and patient scenarios on asthma) | 10 | *↑ Guideline adherence by:* |  |
|  |  |  |  |  |  | ↓ β-blocker and dinitrate combination | + (NS) |
|  |  |  |  |  |  | ↓ Calcium blocker and dinitrate combination | + (NS) |
|  |  |  |  |  |  | ↓ Nitrate, calcium blocker and β-blocker combination | + (NS) |
|  |  |  |  |  |  | ↑ Appropriate use of use of modified release glyceryl trinitrate | 0 |
|  |  |  |  |  |  | ↑ Appropriate use of use of isosorbide mononitrate | 0 |
|  |  |  |  |  |  | ↑ Use of β -blockers | – (NS) |
|  |  |  |  |  |  | ↑ Appropriate use of transdermal glyceryl trinitrate | U |
|  |  |  |  |  |  | ↑ Appropriate use of isosorbide dinitrate | U |
|  |  |  |  |  |  | ↑ Appropriate use of verapamil | U |
|  |  |  |  |  |  | ↑ Appropriate use of diltiazem | U |
|  |  |  |  |  |  | ↑ Appropriate use of short acting glyceryl trinitrate | U |
|  |  |  |  |  |  | ↑ Appropriate use of calcium channel blockers | U |
|  |  |  |  |  |  | ↑ Appropriate use of statins | U |
| Filippi  2003 [18]  Italy  RCT | General practice  *Ambulatory care* | Provider – GPs (n=300)  Patients – CVD prevention in high risk diabetic patients age>30 years (n=15,343) | Reminder activated when physician opened diabetic patients’ EMR. Alert prompted physicians that that patient was at high risk of CVD and to consider anti-platelet drugs. Reminder could be de-activated by physician.  Plus written summary of guidelines.  *System initiated*  *Multi-faceted intervention* | Usual care  Plus written summary of guidelines | 9 | ↑ Antiplatelets | **++** |
| Fretheim  2006 [19]  Norway  RCT | General practices (n=139)  *Ambulatory care* | Provider – GPs (n=501).  Patients – Hypertension or high cholesterol, mostly primary prevention CVD (n=4,152 with prescribing data) | Pop-up reminder triggered at patient’s first visit following elevated BP reading or cholesterol level. Physician reminded to perform computerised cardiovascular risk assessment if patient had not been prescribed treatment. Recommendations on drug choice given. Advice on treatment goals generated for patients on drug therapy.  Plus audit and feedback, academic detailing, written guidelines, patient education materials.  *System initiated*  *Multi-faceted intervention* | Usual care  Plus written guidelines | 10 | ↑ Thiazides | **++** |
|  |  |  |  |  |  | ↑ Thiazides and β-blockers | **++** |
|  |  |  |  |  |  | ↓ Angiotensin II receptor blockers and alpha-blockers | **++** |
| Krall  2004 [20]  US  RCT | HMO  *Ambulatory care* | Provider – primary care clinicians (n=100)  Patient s – Diabetes registry membership and ≥31 years or selected cardiac diagnoses without active aspirin order, aspirin allergy and aspirin contraindications (n=1076) | Alert recommending low dose aspirin therapy linked to patient EMR appeared as intrusive window when predetermined components or eligible screens of patient’s EMR were accessed. The clinician could choose to satisfy or postpone the alert.  *System initiated*  *CDSS only* | Usual care | 7 | ↑ Aspirin use | **++** |
| Montgomery  2000 [51]  UK  RCT | General practices (n=27)  *Ambulatory care* | Provider – GPs (n=74), nurses (n=11)  Patients – Random sample of patients aged 60-80 years with hypertension and receiving anti-hypertensives (n=531) | Computer-based risk calculator (5-year risk of fatal or non-fatal cardiovascular events displayed numerically). Data on risk factors used in calculations (e.g. age, smoking) abstracted from patients’ notes. Paper version presenting identical information about risk also provided.  *User initiated*  *Multi-faceted intervention* | Usual care | 10 | Number of drug classes prescribed | U |
| Murray  2004 [52]  US  RCT | Hospital-based general internal medicine practices (n=4; n=32 practice sessions)  *Institutional care* | Provider – Physicians (general internists, internal medicine residents), pharmacists (n=20)  Patients – Uncomplicated hypertension i.e. no CHD, myocardial infarction, stroke, heart failure, renal insufficiency (n=712) | Care suggestions presenting advice for uncomplicated hypertension management. Suggestions based on data from patient EMR. Computer screen displayed suggested order, possible actions and brief explanation. Physicians could view guidelines and references via the “help” key.  Plus usual CPOE with alerts, written guidelines, didactic group and one-on-one lectures.  *User initiated*  *Multi-faceted intervention* | Usual care  Plus usual CPOE with alerts, written guidelines, didactic group lectures | 10 | Compliance with all anti-hypertensive drug suggestions (n=237) | + (NS) |
|  |  |  |  |  |  | Start or ↑ ACE inhibitor (n=183) | + (NS) |
|  |  |  |  |  |  | Start diuretics (n=113) | – (NS) |
|  |  |  |  |  |  | Start or ↑ calcium channel blocker (n=107) | – (NS) |
|  |  |  |  |  |  | Start or ↑ β-blocker (n=51) | 0 |
| Overhage  1996 [16]  US  RCT | Hospital inpatient (n=6 services; n=24 teams)  *Institutional care* | Provider – General medicine physicians (n=78)  Patients – Received at least 1 preventive care recommendation (n=1,622) | Reminder program analysed data from EMR overnight to identify preventive care measures for eligible patients. Reminder appeared when initiating orders as banner at bottom of computer screen. Physician could display menu of suggested actions and was able to modify these. Paper version also provided.  Plus usual CPOE.  *System initiated*  *Multi-faceted intervention* | Usual care (CPOE) | 10 | ↑ Cholesterol treatment (n=27) | + (NS) |
|  |  |  |  |  |  | ↑ β-blockers (n=24) | – (NS) |
|  |  |  |  |  |  | ↑ Aspirin (n=493) | – (NS) |
|  |  |  |  |  |  | ↑ ACE inhibitors (n=80) | **– –** |
| Roumie  2006 [54]  US  RCT | Veterans Affairs hospital- and community-based practices (n=10)  *Ambulatory care* | Provider – Attending physicians, residents, nurse practitioners, physician assistants (n=182)  Patients – Age 21-90 years, >2 uncontrolled BP measurements in 6 months, taking only 1 anti-hypertensive (n=1,341) | 1. Patient-specific alerts sent by pharmacy through patient EMR. Provider notified of records containing alerts when logged onto a computer. Alert gave brief outline of guideline recommendations, target BP, dates and values of last 3 BP readings, and treatment options.  2. Alerts plus patient education.  Plus link to online guidelines.  *System initiated*  *Multi-faceted intervention* | Usual care  Plus link to online guidelines | 9 | ↑ Diuretics | 1) – (NS)  2) + (NS) |
|  |  |  |  |  |  | ↑ ACE inhibitors or angiotensin receptor blockers | 1) – (NS)  2) + (NS) |
|  |  |  |  |  |  | ↑ Calcium channel blockers | 1) + (NS)  2) + (NS) |
|  |  |  |  |  |  | ↑ β-blockers | 1) – (NS)  2) – (NS) |
|  |  |  |  |  |  | ↑ α-adrenergic antagonist | 1) + (NS)  2) – (NS) |
|  |  |  |  |  |  | Addition of any anti-hypertensive drug | 1) – (NS)  2) + (NS) |
| Sequist  2005 [55]  US  RCT | Community health centres, hospital-based clinics, off-site practices (n=20)  *Ambulatory care* | Provider – Attending physicians and residents (n=194)  Patients – Overdue screening examinations or lack of appropriate medication initiations for CHD and diabetes (n=6,243) | Reminders presenting recommendations for diabetes care and CAD care. Algorithm generated on opening patient record. Determined whether patient had received care in accordance with guidelines. Searched laboratory and radiology results, problem list, medications list, allergy list within EMR. Physicians given option of printing paper version generated using the same algorithm.  *System initiated*  *CDSS only* | Usual care with option of printing paper version of the reminder | 10 | Start aspirin therapy (n=669) | **++** |
|  |  |  |  |  |  | Start statins for LDL cholesterol > 130mg/dL (n=385) | **++** |
|  |  |  |  |  |  | Start β-blocker therapy (n=808) | + (NS) |
|  |  |  |  |  |  | Start ACE inhibitors for diabetics (n=711) | + (NS) |
|  |  |  |  |  |  | Start statins for LDL cholesterol > 130mg/dL in diabetics (n=595) | + (NS) |
| Tierney  2003 [56]  US  RCT | Hospital-based general internal medicine practices (n=4; n=32 practice sessions)  *Ambulatory care* | Provider – Physicians (general internists, fellows, residents; n=94), pharmacists (n=20), nurse practitioner (n=1)  Patients – CHF and/or CHD (n=706) | Care suggestions for CHF and IHD management. Computer-generated suggestions based on data from patient EMR and data entered by physician (vital signs, symptoms, NYHA class). Computer screen displayed suggested order, possible actions and brief explanation. Physicians could view guidelines and references via “help” key.  Plus usual CPOE with alerts, written guidelines, didactic group and one-on-one lectures.  *Mixed*  *Multi-faceted intervention* | Usual care (CPOE with alerts)  Plus written guidelines, didactic group and one-on-one lectures | 10 | Start or ↑ ACE inhibitors (n=216) | + (NS) |
|  |  |  |  |  |  | Start or ↑ β-blockers (n=179) | + (NS) |
|  |  |  |  |  |  | Start or ↑ long-acting nitrates (n=55) | + (NS) |
|  |  |  |  |  |  | Start low dose aspirin (n=155) | – (NS) |
|  |  |  |  |  |  | Start or ↑ diuretics (n=144) | – (NS) |
|  |  |  |  |  |  | Start an anti-hyperlipidemic drug (n=44) | – (NS) |
|  |  |  |  |  |  | Start or ↑ calcium channel blockers (n=38) | – (NS) |
| **Antibiotics** | | | | | | | |
| Flottorp  2002 [47]  Norway  RCT | General practices (n=113)  *Ambulatory care* | Provider – Physicians  Patients – 1) Age>3 years with sore throat (n=12,369)  2) Non-pregnant women aged 16-55 years with UTI (n=5,737) | Pop-up treatment recommendations from evidence based guidelines activated when diagnosis code of 1) sore throat 2) UTI entered into patients’ EMR.  Plus summary of recommendations in poster and electronic format, patient education material, increased telephone consultation fee, workshops, printed material to facilitate discussion, CME points.  *System initiated*  *Multi-faceted intervention* | Usual care (sore throat, UTI guidelines). One intervention controlled the other. | 10 | ↓ Use of antibiotics | 1) **++** |
|  |  |  |  |  |  | ↑ Use of antibiotics | 2) + (NS) |
| Paul  2006 [53]  Germany, Italy, Israel  RCT | Hospital inpatient (n=15 wards)  *Institutional care* | Provider – Physicians (n=unclear)  Patients – Age>18 years with blood cultures drawn, receiving non-prophylaxis antibiotics, systemic inflammatory response syndrome, infection, septic shock, or febrile neutropenia (n=2,326) | Physician inputs variables influencing pathogen probabilities (e.g. demography, vital signs, lab tests, microbiology) and recommendations provided at time of prescribing. Highlights top three regimens with the highest cost-benefit difference. No treatment may also be recommended.  *User initiated*  *CDSS only* | Usual care | 10 | ↑ Appropriate antibiotic treatment | + (NS) |
| **Vaccinations** | | | | | | | |
| Apkon  2005 [42]  US  RCT | Military health practices (n=2)  *Ambulatory care* | Provider – Physicians (n=8), nurse practitioner (n=1), physician assistants (n=3)  Patients – Age>18 years, English speaking with no emergency or obstetric conditions (n=1,902) | Care suggestions after input of medical history and screening information by patients and providers. Information linked to propriety medical database and output detailed diagnosis and treatment options.  *User initiated*  *CDSS only* | Usual care | 8 | Administer pneumococcal vaccination (n=133) | + (NS) |
| Demakis  2000 [21]  US  RCT | Veterans Affairs hospital outpatient centres (n=12)  *Ambulatory care* | Provider – Resident physicians (n=275)  Patients – Eligible for 1 or more standards of care (n=12,989) | Reminder about patients who were suitable for “standards of care review”. Rationale for standard also provided with reminder.  Paper version also provided plus didactic group lecture, written materials.  *System initiated*  *Multi-faceted intervention* | Usual care  Plus didactic group lecture, written materials | 9 | Administer pneumococcal vaccination for patients aged≥65 years or at “high risk” (n=3,447) | **++** |
| Dexter  2001 [17]  US  RCT | Hospital inpatient (n=8 teams)  *Institutional care* | Provider – General medicine residents and medical students (n=202)  Patients – All admitted to general medicine service (n=10,065 admissions for 6,371 patients) | Rule-based reminders generated when the patient’s EMR included at least 1 indication for the selected preventive therapies.  Plus usual CPOE.  *System initiated*  *CDSS only* | Usual care (CPOE) | 10 | ↑ Pneumococcal vaccination (n=1,696) | **++** |
|  |  |  |  |  |  | ↑ Influenza vaccination (n=1,033) | **++** |
| Flanagan  1999 [23]  US  RCT | University hospital and clinics  *Ambulatory care*  *Institutional care* | Provider – staff (n=120) and resident physicians (n=113) and nurses (n=24) | Rule-based recommendations for vaccine orders (age, history, previous vaccination). Provider could choose to over-ride, order recommended or another vaccination.  *System initiated*  *CDSS only* | Usual care | 8 | ↑ Proportion of correct vaccine decisions for tetanus | **++** |
|  |  |  |  |  |  | ↑ Correct vaccine ordered | + (NS) |
| Overhage  1996 [16]  US  RCT | Hospital inpatient (n=6 services; n=24 teams)  *Institutional care* | Provider – General medicine physicians (n=78)  Patients – Received at least 1 preventive care recommendation (n=1,622) | Reminder program analysed data from EMR overnight to identify preventive care measures for eligible patients. Reminder appeared when initiating orders as banner at bottom of computer screen. Physician could display menu of suggested actions and was able to modify these. Paper version also provided.  Plus usual CPOE.  *System initiated*  *Multi-faceted intervention* | Usual care (CPOE) | 10 | ↑ Pneumococcal vaccination (n=514) | + (NS) |
| Safran  1995 [22]  US  RCT | Hospital-based general medicine practices (n=5)  *Ambulatory care* | Provider – Resident and staff physicians (n=126), nurse practitioners (n=10).  Patients – HIV (n=349) | Alerts sent automatically to provider about important event (e.g. lab results out of normal range) and reminder posted in patient EMR (e.g. vaccination due). Providers could act upon the alert, indicate alert was inappropriate or not applicable, or indicate patient refused recommendation.  *System initiated*  *CDSS only* | Usual care (access to library and CME seminars) | 9 | Administer pneumococcal vaccination (n=104) | **++** |
|  |  |  |  |  |  | Administer influenza vaccination (n=119) | **++** |
|  |  |  |  |  |  | Administer tetanus vaccination (n=120) | + (NS) |
| Tang 1999  US [24]  Quasi-experimental | Internal medicine medical centre  *Ambulatory care* | Provider – Clinicians (n=34)  Patients – 65 years and over with one or more non-acute clinic visit during influenza season (n=1885 with 3117 visits) | Rule-based clinician reminder for vaccination when provider opened patient chart. Provider could comply by ordering vaccination, documenting counselling was performed, documenting vaccine was offered but declined or patient received vaccination elsewhere.  *System initiated*  *CDSS only* | Usual care | 4 | ↑ Influenza vaccinations | **++** |
| Tierney  2005 [34]  US  RCT | Hospital-based general internal medicine practices (n=4)  *Ambulatory care* | Provider – Physicians (general internists, internal medicine or medicine-paediatric residents; n=274), pharmacists (n=20)  Patients – Age≥18 years with asthma or COPD (n=699) | Care suggestions for asthma and COPD management. Computer-generated suggestions based on data from patient EMR and data entered by physician (vital signs, symptoms, NYHA class). Computer screen displayed suggested order, possible actions and brief explanation. Physicians could view guidelines and references via “help” key.  Plus usual CPOE with alerts, written guidelines, didactic group and one-on-one lectures.  *Mixed*  *Multi-faceted intervention* | Usual care (CPOE with alerts)  Plus written guidelines, didactic group and one-on-one lectures | 10 | Administer influenza vaccination (n=177) | – (NS) |
|  |  |  |  |  |  | Administer pneumococcal vaccination (n=167) | – (NS) |
| Tierney  2003 [56]  US  RCT | Hospital-based general internal medicine practices (n=4; n=32 practice sessions)  *Ambulatory care* | Provider – Physicians (general internists, fellows, residents; n=94), pharmacists (n=20), nurse practitioner (n=1)  Patients – CHF and/or CHD (n=706) | Care suggestions for CHF and IHD management. Computer-generated suggestions based on data from patient EMR and data entered by physician (vital signs, symptoms, NYHA class). Computer screen displayed suggested order, possible actions and brief explanation. Physicians could view guidelines and references via “help” key.  Plus usual CPOE with alerts, written guidelines, didactic group and one-on-one lectures.  *Mixed*  *Multi-faceted intervention* | Usual care (CPOE with alerts)  Plus written guidelines, didactic group and one-on-one lectures | 10 | Administer pneumococcal vaccination (n=186) | + (NS) |
| **Respiratory** | | | | | | | |
| Eccles  2002 [45]  UK  RCT | General practices (n=60)  *Ambulatory care* | Provider – GPs (4.6 partners per practice) and practice nurses. Single-handed practices excluded.  Patients – Age>18 years with asthma (n=2,776 with prescribing data). | Information in patients’ EMR triggered guideline and presentation of patient scenarios on asthma. System offered management suggestions and requested entry of relevant information to be stored in EMR.  *System initiated*  *CDSS only* | Usual care (computerised guideline and patient scenarios on angina) | 10 | *↑ Guideline adherence by:*  ↑ Appropriate use of short acting β2 agonists | U |
|  |  |  |  |  |  | ↑ Appropriate use of inhaled corticosteroids | U |
|  |  |  |  |  |  | ↑ Appropriate use of long acting β2 agonists | U |
|  |  |  |  |  |  | ↑ Appropriate use of oral steroids | U |
|  |  |  |  |  |  | ↑ Appropriate use of oral bronchodilators | U |
| Tierney  2005 [34]  US  RCT | Hospital-based general internal medicine practices (n=4)  *Ambulatory care* | Provider – Physicians (general internists, internal medicine, medicine-paediatric residents; n=274), pharmacists (n=20).  Patients – Age≥18 years with asthma, COPD, emphysema or had received ≥2 respiratory medications (n=706). | Care suggestions for asthma and COPD management. Computer-generated suggestions based on data from patient EMR and data entered by physician (vital signs, symptoms, NYHA class). Computer screen displayed suggested order, possible actions and brief explanation. Physicians could view guidelines and references via “help” key.  Plus usual CPOE with alerts, written guidelines, didactic group and one-on-one lectures.  *Mixed*  *Multi-faceted intervention* | Usual care (CPOE with alerts)  Plus written guidelines, didactic group and one-on-one lectures) | 10 | Start Ipratropium (n=138) | + (NS) |
|  |  |  |  |  |  | Switch to cheaper β-agonist (n=54) | + (NS) |
|  |  |  |  |  |  | Start oral corticosteroids (n=19) | + (NS) |
|  |  |  |  |  |  | Start inhaled β-agonist (n=63) | – (NS) |
|  |  |  |  |  |  | Start inhaled corticosteroids (n=27) | 0 |
| **Anticoagulant Therapy** | | | | | | | |
| Dexter  2001 [17]  US  RCT | Hospital inpatient (n=8 teams)  *Institutional care* | Provider – General medicine residents and medical students (n=202)  Patients – All admitted to general medicine service (n=10,065 admissions for 6,371 patients) | Rule-based reminders generated when the patient’s EMR included at least one indication for the selected preventive therapies.  Plus usual CPOE.  *System initiated*  *CDSS only* | Usual care (CPOE) | 10 | ↑ Subcutaneous heparin (n=1,083) | **++** |
| Kucher  2005 [49]  US  RCT | Hospital inpatient  *Institutional care* | Provider – Physicians (n=120)  Patient – Admitted to medical and surgical wards, age>18 years, at increased risk of thromboembolisms (n=2,506) | Program used eight common risk factors to identify patients at increased risk of venous thromboembolism (cumulative risk score >4 and no prophylaxis). Provider required to acknowledge alert and withhold or order prophylaxis. Computer alert linked to online hospital guidelines.  *System initiated*  *CDSS only* | Usual care  Plus online guidelines | 8 | ↑ Pharmacological or mechanical prophylaxis | **++** |
|  |  |  |  |  |  | ↑ Unfractionated heparin | **++** |
|  |  |  |  |  |  | ↑ Enoxaparin | + (NS) |
|  |  |  |  |  |  | ↑ Warfarin | – (NS) |
| Overhage  1996 [16]  US  RCT | Hospital inpatient (n=6 services; n=24 teams)  *Institutional care* | Provider – General medicine physicians (n=78)  Patients – Received at least 1 preventive care recommendation (n=1,622) | Reminder program analysed data from EMR overnight to identify preventive care measures for eligible patients. Reminder appeared when initiating orders as banner at bottom of computer screen. Physician could display menu of suggested actions and was able to modify these. Paper version also provided.  Plus usual CPOE.  *System initiated*  *Multi-faceted intervention* | Usual care (CPOE) | 10 | ↑ Heparin prophylaxis (n=58) | + (NS) |
| **Osteoporosis** | | | | | | | |
| Feldstein  2006 [46]  US  RCT | HMO (n=15 primary care clinics)  *Ambulatory care* | Provider – Primary care providers (n=159)  Patients – Female patients aged 50-89 years, suffered a fracture in 1999, not received BMD measurement or medication for osteoporosis (n=311) | 1. EMR-based email message from chairman of quality-improvement committee informing provider of patient’s risk of osteoporosis (listing internal and external guideline resources). Reminder sent 3 months later if BMD or medication for osteoporosis had not been ordered.  2. Email message plus reminder mailed to patients.  *System initiated*  *Multi-faceted intervention* | Usual care | 8 | ↑ Osteoporosis medication | 1) **++**  2) **++** |
| Overhage  1996 [16]  US  RCT | Hospital inpatient (n=6 services; n=24 teams)  *Institutional care* | Provider – General medicine physicians (n=78)  Patients – Received at least 1 preventive care recommendation (n=1,622) | Reminder program analysed data from EMR overnight to identify preventive care measures for eligible patients. Reminder appeared when initiating orders as banner at bottom of computer screen. Physician could display menu of suggested actions and was able to modify these. Paper version also provided.  Plus usual CPOE.  *System initiated*  *Multi-faceted intervention* | Usual care (CPOE) | 10 | ↑ Oestrogen treatment post-menopause (n=475) | + (NS) |
|  |  |  |  |  |  | ↑ Calcium treatment for osteopenia (n=475) | + (NS) |
| **Other Clinical Areas** | | | | | | | |
| Kralj  2003 [48]  US  RCT | Community oncology practices (n=2)  *Ambulatory care* | Provider – Physicians (n=unknown)  Patient – Cancer patients with anaemia (n=2,170) | Pop-up reminder to prescribe erythropoietin to patients with Hgb<12g/dL in the last 14 days and not currently receiving erythropoietin.  *System initiated*  *CDSS only* | Usual care | 10 | ↑ Erythropoietin for low (<12g/dL) Hgb patients | **++** |
| Kuperman  1999 [50]  US  RCT | Hospital inpatient  *Institutional care* | Provider – 63, 7 and 22 alerts reviewed by physician, nurse, and phone operator respectively in intervention group.  Patients – Medical and surgical inpatients (n=192 alerts for 178 patients) | Continuously running event monitor determining whether new patient data satisfies rule-based alerting criteria. 12 alerting rules (single laboratory result, changes in laboratory results over time, drug-laboratory interaction). Program automatically pages physician who logs onto a computer to view alert. Review screen displays patient details, alert, active medications related to alert, and actions to be undertaken. Included a fail-safe notification system if physician does not respond to the alert (nurse notification followed by phone operator).  *System initiated*  *Multi-faceted intervention* | Usual care (laboratory telephoned unit secretary or nurse) | 6 | ↓ Time until appropriate treatment ordered (e.g. potassium, insulin, drugs for high potassium levels) | **++** |
| Safran  1995 [22]  US  RCT | Hospital-based general medicine practices (n=5)  *Ambulatory care* | Provider – Resident and staff physicians (n=126), nurse practitioners (n=10).  Patients – HIV (n=349) | Alerts sent automatically to provider about important event (e.g. lab results out of normal range) and reminder posted in patient EMR (e.g. vaccination due). Providers could act upon the alert, indicate alert was inappropriate or not applicable, or indicate patient refused recommendation.  *System initiated*  *CDSS only* | Usual care (access to library and CME seminars) | 9 | ↑ PCP prophylaxis (n=41) | **++** |
|  |  |  |  |  |  | Start Zidovudine or didanosine (n=34) | + (NS) |

* Unless otherwise stated, number of patients is close to or equal to that specified in the “participants” column, or was not reported.

+ (NS) indicates intervention favoured the CDSS but was not statistically significant; – (NS) indicates intervention favoured comparison group but was not statistically significant; 0 = no difference between groups; **++** indicates intervention favoured CDSS and was statistically significant; **- -** indicates intervention favoured comparator and was statistically significant; U = unclear.

ACE = angiotensin-converting enzyme; BMD = bone mineral density; BP = blood pressure; CAD = coronary artery disease; CDSS = computerised clinical decision support system; CHD = coronary heart disease; CHF = congestive heart failure; CME = continuing medical education; CPOE = computerised provider order entry; COPD = chronic obstructive pulmonary disease; CVD = cardiovascular disease; EMR = electronic medical record; GI = gastro intestinal; GP = general practitioner; Hgb = haemoglobin; HIV = human immuno-deficiency virus; HMO = Health Maintenance Organisation; IHD = ischemic heart disease; LDL = low-density lipoprotein; MI = myocardial infarction; NSAIDs = non-steroidal anti-inflammatory drugs; NYHA = New York Heart Association; PCP = P carinii pneumonia; RCT = randomised controlled trial ; UTI = urinary tract infection;
