# Supplementary material for: Do computerised clinical decision support systems for prescribing change practice? A systematic review of the literature (1990-2007)
Source: BMC Health Serv Res. 2009 Aug 28;9:154. doi: 10.1186/1472-6963-9-154 (PMC2744674; doi:10.1186/1472-6963-9-154)
Supplement: Additional file 5 — Table S4: Key study features and results (Initiating treatment - After drug selection). * Unless otherwise stated, number of patients is close to or equal to that specified in the "participants" column, or was not reported. + (NS) indicates intervention favoured the CDSS but was not statistically significant; - (NS) indicates intervention favoured comparison group but was not statistically significant; 0 = no difference between groups; ++ indicates intervention favoured CDSS and was statistically significant; - - indicates intervention favoured comparator and was statistically significant; U = unclear. CDSS = computerised clinical decision support system; CPOE = computerised provider order entry; COPD = chronic obstructive pulmonary disease; CNS = central nervous system; CVD = cardiovascular disease; ED = emergency department; EMR = electronic medical record; GP = general practitioner; HMO = Health Maintenance Organisation; ICU = intensive care unit; IV = intravenous; MRSA = methicillin-resistant Staphylococcus aureus; NSAIDs = non-steroidal anti-inflammatory drugs; RCT = randomised controlled trial; TCA = tertiary amine tricyclics antidepressant. [file 1472-6963-9-154-S5.doc]

**Table III: Key study features and results (Initiating treatment – After drug selection**)

| **Study** | **Setting** | **Participants** | **Intervention** | **Comparison** | **QA Score (N/10)** | **Practice change in line with intent of CDSS *** | **Change in Performance** |
| --- | --- | --- | --- | --- | --- | --- | --- |
| **Cardiovascular** | | | | | | | |
| Martens  2007 [57]  Netherlands  RCT | General practices (n=14)  *Ambulatory care* | Provider – GPs (n=53)  Patients – 180 to 2,373 patients per GP on average with high cholesterol. | Provider required to input patient’s diagnosis and disease severity. At time of prescribing, system checked drug choice against age, gender and diagnosis information and determined if prescribing was best practice and reminder was triggered if outside recommendations. Reminders suggested alternative drugs, doses, durations and administration or not to prescribe.  *System initiated*  *CDSS only* | Usual care (asthma, COPD and antibiotic reminders) | 9 | ↓ Statins for newly diagnosed patients with diabetes or CVD age 18-70 years with cholesterol <3.5mmol | + (NS) |
|  |  |  |  |  |  | ↑ Statins for newly diagnosed patients with diabetes or CVD age 18-70 years with cholesterol >5.5mmol | + (NS) |
| **Antibiotics** | | | | | | | |
| Davis  2007 [61]  US  RCT | Primary care paediatric clinics (n=2)  *Ambulatory care* | Provider – Resident and attending physicians, nurse practitioners (n=44)  Patients – Paediatric patients with otitis media , allergic rhinitis, sinusitis, constipation, pharyngitis, croup, urticaria, broncholitis (n=12,195 patient visits). | Alert detailing evidence supporting or refuting current choice of medication, indication, or duration of therapy. Links provided to a pdf version of article or abstract from which the evidence was derived.  *System initiated*  *CDSS only* | Usual care | 7 | ↓ Antibiotics for otitis media | **++** |
|  |  |  |  |  |  | ↑ Amoxicillin for otitis media | + (NS) |
|  |  |  |  |  |  | Correct antibiotic dosage | – (NS) |
|  |  |  |  |  |  | ↓ Duration of antibiotics (<10 days) | – (NS) |
| Christakis 2001  US [25]  RCT | Primary care paediatric clinics  *Ambulatory care* | Provider – Physicians (n=7), nurse practitioners (n=2)  Patients - Patients with otitis media (n=14, 414 visits, 1139 visits for acute otitis media) | Pop-up screen supporting or refuting providers’ selection of antibiotic, indication, and duration of therapy. Links provided to a pdf version of article or abstract from which the evidence was derived.  *System initiated*  *CDSS only* | Usual care | 8 | ↓ Duration of antibiotic therapy | **++** |
|  |  |  |  |  |  | ↓Number of patients treated with antibiotics | **–**  (NS) |
| Hulgan  2004 [26]  US  Quasi-experimental | Hospital inpatient  *Institutional care* | Provider – Physicians, nurses, nurse practitioners, pharmacists (77% of orders placed by physicians)  Patients – Inpatients (n=7,623 orders) | Advice initiated when IV quinolones were ordered. Patients’ active orders searched to identify if eligible for oral medication (taking other oral medications or on solid diet). If eligible providers asked to consider oral medication. Provider had to indicate reasons for continuing with IV order.  Plus usual CPOE.  *System initiated*  *CDSS only* | Usual care (CPOE) | 4 | ↑ Oral quinolones instead of IV quinolones | **++** |
| Martens  2007 [57]  Netherlands  RCT | General practices (n=23)  *Ambulatory care* | Provider – GPs (n=53)  Patients – 1,180 to 2,373 patients per GP on average. | Provider required to input patient’s diagnosis and disease severity. At time of prescribing, system checked drug choice against age, gender and diagnosis information and determined if prescribing was best practice and reminder was triggered if outside recommendations. Reminders suggested alternative drugs, doses, durations and administration or not to prescribe.  *System initiated*  *CDSS only* | Usual care (cholesterol reminders) | 9 | ↑Trimethoprim, nitrofurantoin for cystitis | **++** |
|  |  |  |  |  |  | ↓ Quinolones for cystitis | **++** |
|  |  |  |  |  |  | ↓ Phenethicillin, azithromycin, phenoxymethyl penicillin for sore throat | **++** |
|  |  |  |  |  |  | ↓ Doxycycline and amoxicillin for bronchitis | + (NS) |
|  |  |  |  |  |  | ↓ Systemic use of antibiotics for sore throat without doxycycline for acute sinusitis | + (NS) |
|  |  |  |  |  |  | ↓ Amoxicillin, azithromycin for otitis media | + (NS) |
|  |  |  |  |  |  | ↓ Systemic use of antibiotics for otitis media | + (NS) |
|  |  |  |  |  |  | ↑ Minocycline, benzoyl peroxide, salicylic acid for acne | + (NS) |
|  |  |  |  |  |  | ↑ Phenoxymethyl penicillin, phenethicillin, erythromycin for erysipelas | + (NS) |
|  |  |  |  |  |  | ↑ Fusidic acid, zinc with disinfectant for impetigo | + (NS) |
|  |  |  |  |  |  | ↑ Flucloxacillin, azithromycin for impetigo | + (NS) |
|  |  |  |  |  |  | ↓ Systemic use of antibiotics for sore throat | + (NS) |
|  |  |  |  |  |  | ↓ Doxycycline for sinusitis | + (NS) |
|  |  |  |  |  |  | ↑ Benzoyl peroxide, salicylic acid for acne | – (NS) |
|  |  |  |  |  |  | ↑ Erythromycin, minocycline, cyproterone acetate for acne | – (NS) |
|  |  |  |  |  |  | ↑ Co-trimoxazole, ciprofloxacin, norfloxacin for prostatitis | – (NS) |
| Madaras-Kelly  2006 [27]  US  Quasi-experimental | Veterans Affairs hospital  *Institutional care* | Provider – Physicians (n=unknown)  Patients – MRSA, various infections (n=80 infections) | Prompt inserted next to fluoroquinolone selections on CPOE screen asking provider to prescribe alternative agent. Subsequent screen asked providers to confirm need for fluoroquinolone if it was still ordered.  Plus usual CPOE.  *System initiated*  *CDSS only* | Usual care (CPOE) | 4 | ↓ Fluoroquinolones | **++** |
| Shojania  1998 [28]  US  RCT | Hospital inpatient  *Institutional care* | Provider – Physicians (n=396)  Patients – Inpatients (n=1,798) | Screen appeared when provider initiated IV vancomycin or when vancomycin continued beyond 72 hours. Provider required to enter indication for use (with free text to describe “other” indications) or abort order or justify continuation. Guidelines also presented on this screen.  Plus usual CPOE.  *System initiated*  *CDSS only* | Usual care (CPOE) | 7 | ↓ IV vancomycin | **++** |
|  |  |  |  |  |  | ↓ Duration of vancomycin therapy | + (NS) |
| **Respiratory** | | | | | | | |
| Davis  2007 [61]  US  RCT | Primary care paediatric clinics (n=2)  *Ambulatory care* | Provider – Resident and attending physicians, nurse practitioners (n=44)  Patients – Paediatric patients with allergic rhinitis, sinusitis, constipation, pharyngitis, croup, urticaria, broncholitis, otitis media (n=12,195 patient visits). | Alert detailing evidence supporting or refuting current choice of medication, indication, or duration of therapy. Links provided to a pdf version of article or abstract from which the evidence was derived.  *System initiated*  *CDSS only* | Usual care | 7 | Proper choice of treatment (overall, including antibiotics) | **++** |
|  |  |  |  |  |  | ↓ Loratadine for allergic rhinitis | **++** |
|  |  |  |  |  |  | Proper choice of treatment (sinusitis, pharyngitis, croup, constipation, urticaria combined) | + (NS) |
|  |  |  |  |  |  | Appropriate use of salbutamol for bronchiolitis | U |
| Kuilboer  2006 [58]  Netherlands  RCT | General practices (n=32)  *Ambulatory care* | Provider – GPs (n=40)  Patients – Age>0 years with asthma, chronic bronchitis, emphysema or other chronic pulmonary diseases (n=156,772 enrolled, approximately 10% had asthma or COPD) | System reviews and critiques treatment of asthma/ COPD patients. Generates feedback about choices, transforms clinical measurements (e.g. peak-flow) and makes recommendations (provider can request additional information).  *System initiated*  *CDSS only* | Usual care | 10 | ↓ Antihistamines (age 0-11, 12-39, 40-59, >60 years) | 0, 0, + (NS), 0 |
|  |  |  |  |  |  | ↓ Cromoglycate to children with intolerance to inhaled corticosteroids or adults with allergic asthma (age 0-11, 12-39, 40-59, >60 years) | 0, **++**, 0, 0 |
|  |  |  |  |  |  | ↓ Deptropine in children | + (NS) |
|  |  |  |  |  |  | ↓ Oral bronchodilators in children | – (NS) |
|  |  |  |  |  |  | Appropriate use of oral corticosteroids | U |
| Martens  2007 [57]  Netherlands  RCT | General practices (n=14)  *Ambulatory care* | Provider – GPs (n=53)  Patients – 180 to 2,373 patients per GP on average with asthma or COPD. | Provider required to input patient’s diagnosis and disease severity. At time of prescribing, system checked drug choice against age, gender and diagnosis information and determined if prescribing was best practice and reminder was triggered if outside recommendations. Reminders suggested alternative drugs, doses, durations and administration or not to prescribe.  *System initiated*  *CDSS only* | Usual care (cholesterol reminders) | 9 | ↓ Inhaled corticosteroids for newly diagnosed COPD patients age>40 years | **++** |
|  |  |  |  |  |  | ↑ Budesonide, fluticasone for mildly persistent asthma | + (NS) |
|  |  |  |  |  |  | ↓ Prescriptions for intermittent asthma | + (NS) |
|  |  |  |  |  |  | ↑ Terbutaline, salbutamol for intermittent-moderately persistent asthma (acute symptoms) | – (NS) |
|  |  |  |  |  |  | ↑ Budesonide, fluticasone and salmeterol or eformoterol for severe persistent asthma | – (NS) |
|  |  |  |  |  |  | ↑ Ipratropium bromide salbutamol for newly diagnosed COPD patients age>40 years | 0 |
| **Elderly** | | | | | | | |
| Judge  2006 [59]  US  RCT | Long-term care units (n=7)  *Institutional care* | Provider – Physicians, nurse practitioners, physician assistants (n=27)  Patients – Residents of long-term care units (n=4,282 alerts) | Alert generated when entering drug orders if order involved: high-severity drug interactions; was for patient with abnormal laboratory result; monitoring for potential adverse effects; prophylactic measures to address potential adverse effects; dose ranges to reduce adverse effects in the elderly (41 different alerts in total). Alerts included instructions for laboratory monitoring and recommendations for reconsidering drug orders and monitoring for possible side-effects.  Plus usual CPOE.  *System initiated*  *CDSS only* | Usual care (CPOE) | 8 | Appropriate action taken (overall) | + (NS) |
|  |  |  |  |  |  | *Appropriate action taken for:* |  |
|  |  |  |  |  |  | Warfarin orders (n=517) | **++** |
|  |  |  |  |  |  | Dose recommendations (n=395) | + (NS) |
|  |  |  |  |  |  | Drug interactions (n=72) | + (NS) |
|  |  |  |  |  |  | Drugs with CNS side-effects (e.g. long-acting benzodiazepines) (n=874) | + (NS) |
|  |  |  |  |  |  | Related to multiple anti-platelet orders (n=69) | – (NS) |
|  |  |  |  |  |  | Anti-cholinergic side-effects (n=128) | – (NS) |
|  |  |  |  |  |  | Drugs with constipation side-effects (e.g. opioid therapy) (n=578) | – (NS) |
| Peterson  2007 [29]  US  RCT | Hospital (ED, ICU, sub-acute unit)  *Institutional care* | Provider – Physicians (n=778)  Patients – Age≥65 years (n=2,981) | Guided dosing system prompted physicians about appropriate initial dosing for sedatives, neuroleptics, anti-emetics and skeletal muscle relaxants for common indications. It discouraged prescribing of contraindicated drugs. Prompts displayed with study-related dosing information and communicated titration strategies, possible adverse effects and key monitoring parameters.  *System initiated*  *CDSS only* | Usual care | 6 | Acceptance rate of recommended doses (e.g. antihistamines, anti-emetics, benzodiazepines, anti-spasmodics) | **++** |
| Smith  2006 [30]  US  Quasi-experimental | HMO (n=15 primary care clinics)  *Ambulatory care* | Provider – Family practitioners, internal medicine physicians (n=152), nurse practitioners (n=25), physician assistants (n=32)  Patients – Received a new medication (n=unclear) | Alert generated when provider ordered non-preferred agent cautioning against prescribing certain medications in the elderly. Presented alternative medication. Prominent warnings regarding falls and fractures.  Plus usual CPOE  *System initiated*  *CDSS only* | Usual care (CPOE) | 4 | ↑ Short-acting benzodiazepines, secondary TCAs (age<65, age>65 years) | **++**, 0 |
|  |  |  |  |  |  | ↓ Long-acting benzodiazepines and tertiary TCAs (age<65, age>65 years) | 0, **++** |
|  |  |  |  |  |  | ↑ Use of nortriptyline instead of amitriptyline | **++** |
| Tamblyn  2003 [36]  Canada  RCT | General practice  *Ambulatory care* | Provider – GPs age>30 years with minimum of 100 elderly patients and 70% of time fee-for-service (n=107)  Patients – Age≥66 years (n=12,560) | Alert identified 159 clinically relevant prescribing problems in the elderly (drug-disease contraindications, drug interactions, drug-age contraindications, duration of therapy, therapeutic duplication). Alerts appeared when patients’ EMR was opened, prescription records downloaded, and when physician updated EMR with patients’ health problem and prescriptions. Alert identified the nature of the problem, possible consequences and alternatives.  *System initiated*  *CDSS only* | Usual care | 9 | ↓ Inappropriate prescriptions (e.g. NSAIDs, benzodiazepines) | **++** |
|  |  |  |  |  |  | ↓ Excess duration of therapy | **++** |
|  |  |  |  |  |  | ↓ Drug-age contraindication | + (NS) |
|  |  |  |  |  |  | ↓ Drug-disease contraindication | + (NS) |
|  |  |  |  |  |  | ↓ Therapeutic duplication | + (NS) |
|  |  |  |  |  |  | ↓ Drug interaction | - (NS) |
| **Other Clinical Areas** | | | | | | | |
| Davis  2007 [61]  US  RCT | Primary care paediatric clinics (n=2)  *Ambulatory care* | Provider – Resident and attending physicians, nurse practitioners (n=44)  Patients – Paediatric patients with allergic rhinitis, sinusitis, constipation, pharyngitis, croup, urticaria, broncholitis, otitis media (n=12,195 patient visits). | Alert detailing evidence supporting or refuting current choice of medication, indication, or duration of therapy. Links provided to a pdf version of article or abstract from which the evidence was derived.  *System initiated*  *CDSS only* | Usual care | 7 | Proper choice of treatment (overall, including antibiotics) | **++** |
|  |  |  |  |  |  | Proper choice of treatment (sinusitis, pharyngitis, croup, constipation, urticaria combined) | + (NS) |
| Feldstein  2006 [60]  US  Quasi-experimental | HMO (n=15 primary care clinics)  *Ambulatory care* | Provider – Physicians, nurse practitioners, physician assistants (n=236)  Patients – Receiving warfarin (n=4743) | Drug interaction alert (drugs interacting with warfarin). Included a short description of clinical issue and suggested alternatives.  *System initiated*  *CDSS only* | Usual care | 4 | ↓ Medications interacting with warfarin (NSAIDs, acetaminophen, fluconazole, metronidazole, sulfamethoxazole) | **++** |

* Unless otherwise stated, number of patients is close to or equal to that specified in the “participants” column, or was not reported.

+ (NS) indicates intervention favoured the CDSS but was not statistically significant; – (NS) indicates intervention favoured comparison group but was not statistically significant; 0 = no difference between groups; **++** indicates intervention favoured CDSS and was statistically significant; **- -** indicates intervention favoured comparator and was statistically significant; U = unclear.

CDSS = computerised clinical decision support system; CPOE = computerised provider order entry; COPD = chronic obstructive pulmonary disease; CNS = central nervous system; CVD = cardiovascular disease; ED = emergency department; EMR = electronic medical record; GP = general practitioner; HMO = Health Maintenance Organisation; ICU = intensive care unit; IV = intravenous; MRSA = methicillin-resistant *Staphylococcus aureus*; NSAIDs = non-steroidal anti-inflammatory drugs; RCT = randomised controlled trial ; TCA = tertiary amine tricyclics antidepressant.
