# Supplementary material for: Do computerised clinical decision support systems for prescribing change practice? A systematic review of the literature (1990-2007)
Source: BMC Health Serv Res. 2009 Aug 28;9:154. doi: 10.1186/1472-6963-9-154 (PMC2744674; doi:10.1186/1472-6963-9-154)
Supplement: Additional file 6 — Table S5: Key study features and results (Monitoring existing therapy). * Unless otherwise stated, number of patients is close to or equal to that specified in the "participants" column, or was not reported. + (NS) indicates intervention favoured the CDSS but was not statistically significant; - (NS) indicates intervention favoured comparison group but was not statistically significant; 0 = no difference between groups; ++ indicates intervention favoured CDSS and was statistically significant; - - indicates intervention favoured comparator and was statistically significant; U = unclear. ACE = angiotensin-converting enzyme; BP = blood pressure; ALT = alanine aminotransferase; AST = aspartate aminotransferase; CBC = complete blood count; CDSS = computerised clinical decision support system; CHD = coronary heart disease; CME = continuing medical education; CPOE = computerised provider order entry; COPD = chronic obstructive pulmonary disease; EMR = electronic medical record; FSH = follicle-stimulating hormone; HIV = human immuno-deficiency virus; HMO = health maintenance organisation; INR = international normalised ratio; IV = intravenous; LFT = liver function test; LH = luteinizing hormone; NYHA = New York Heart Association; PTR = prothrombin time ratio; RCT = randomised controlled trial; TSH = thyroid stimulating hormone; [file 1472-6963-9-154-S6.doc]

**Table IV: Key study features and results (Monitoring existing therapy**)

| **Study** | **Setting** | **Participants** | **Interventions** | **Comparison** | **QA Score (N/10)** | **Practice change in line with intent of CDSS *** | **Change in Performance** |
| --- | --- | --- | --- | --- | --- | --- | --- |
| **Maintenance Prescribing** | | | | | | | |
| **Cardiovascular Disease** | | | | | | | |
| Lester  2006 [67]  US  RCT | Primary care practice  *Ambulatory care* | Provider – Physicians who used EMR for >80% of visits (n=14)  Patients – Age>30 years with CHD or at risk of CHD and elevated cholesterol levels (n=235) | Customised email for each patient informing physician of elevated and outdated cholesterol level results. Ranked drug choices (based algorithm incorporating goals, predicted post-intervention cholesterol result, patient insurance and co-pay status). Provider could change; repeat or decline advice. Alert occurred outside consultation but linked to EMR.  *System initiated*  *CDSS only* | Usual care | 8 | Change statin prescription within 1 month (i.e. substitution or dose change) | **++** |
|  |  |  |  |  |  | Change statin prescription within 12 months (i.e. substitution or dose change) | + (NS) |
| Roumie  2006 [54]  US  RCT | Veterans Affairs hospital- and community-based clinics (n=10)  *Ambulatory care* | Provider – Attending physicians, residents, nurse practitioners, physician assistants (n=182)  Patients – Age 21-90 years, >2 uncontrolled BP measurements in 6 months, taking only 1 anti-hypertensive (n=1,341) | 1. Patient-specific alerts sent by pharmacy through patient EMR. Provider notified of records containing alerts when logged onto a computer. Alert gave brief outline of guideline recommendations, target BP, dates and values of last 3 BP readings, and treatment options.  2. Alerts plus patient education.  Plus link to online guidelines.  *System initiated*  *Multi-faceted intervention* | Usual care  Plus link to online guidelines | 9 | ↑ Dose of anti-hypertensive drugs | 1) – (NS)  2) – (NS) |
| **Respiratory** | | | | | | | |
| Tierney  2005 [34]  US  RCT | Hospital-based general internal medicine practices (n=4)  *Ambulatory care* | Provider – Physicians (general internists, internal medicine, medicine-paediatric residents; n=274), pharmacists (n=20).  Patients – Age≥18 years with asthma, COPD, emphysema or had received ≥2 respiratory medications (n=706). | Care suggestions for asthma and COPD management. Suggestions based on data from patient EMR and data entered by physician (vital signs, symptoms, NYHA class). Computer screen displayed suggested order, possible actions and brief explanation. Physicians could view guidelines and references via “help” key.  Plus usual CPOE with alerts, written guidelines, didactic group and one-on-one lectures.  *Mixed*  *Multi-faceted intervention* | Usual care (CPOE with alerts)  Plus written guidelines, didactic group and one-on-one lectures | 10 | ↑ or ↓ Theophylline dose (n=63) | 0 |
| McCowan  2001 [69]  UK  RCT | General practices (n=17)  *Ambulatory care* | Provider – Physicians (3.4 to 3.8 partners per practice)  Patients – Random sample of patients from the asthma register in each practice (n=477) | Physician conducted review of intervention patients. System provided reminders on asthma management presented during consultation. Software included bank of clinical scenarios, constructive feedback, and prediction of future morbidity (by matching patients to cases in database). Included facility to print customised self-management plans and patient advice sheets.  *User initiated*  *CDSS only* | Usual care | 7 | Maintenance prescribing (e.g. increase dose preventive inhaler) | U |
| **Other Clinical Areas** | | | | | | | |
| Safran  1995 [22]  US  RCT | Hospital-based general medicine practices (n=5)  *Ambulatory care* | Provider – Resident and staff physicians (n=126), nurse practitioners (n=10).  Patients – HIV (n=349) | Alerts sent automatically to provider about important event (e.g. lab results out of normal range) and reminder posted in patient EMR (e.g. vaccination due). Providers could act upon the alert, indicate alert was inappropriate or not applicable, or indicate patient refused recommendation.  *System initiated*  *CDSS only* | Usual care (access to library and CME seminars) | 9 | Change zidovudine dose (n=36) | + (NS) |
| **Laboratory Testing** | | | | | | | |
| **Cardiovascular Disease** | | | | | | | |
| Palen  2006 [71]  US  RCT | HMO (n=16 primary care clinics)  *Ambulatory care* | Provider – Internal medicine and family practice physicians (n=207).  Patients – Received prescription for targeted medication (n=26,586). | Non-intrusive alerts. List of medications in CPOE system. Physician orders drug and for selected medications screen automatically includes reminder that laboratory test is required.  Plus usual CPOE and academic detailing.  *System initiated*  *Multi-faceted intervention* | Usual care (CPOE and custom formulary) | 8 | ↑ Laboratory monitoring rates for: |  |
|  |  |  |  |  |  | Gemfibrozil (ALT or AST) (n=1,023) | **++** |
|  |  |  |  |  |  | Statins (ALT or AST) (8,962) | + (NS) |
|  |  |  |  |  |  | Niacin (ALT or AST, glucose, uric acid) (n=70) | + (NS) |
|  |  |  |  |  |  | Digoxin (creatinine, potassium, digoxin concentration) (n=420) | + (NS) |
|  |  |  |  |  |  | ACE inhibitors (creatinine, potassium) (n=5,828) | – (NS) |
|  |  |  |  |  |  | Diuretic (creatinine, potassium) (n=9,654) | – (NS) |
|  |  |  |  |  |  | Losartan potassium (creatinine, potassium) (n=939) | – (NS) |
|  |  |  |  |  |  | Potassium chloride (creatinine, potassium) (n=2,914) | – (NS) |
| **Respiratory** | | | | | | | |
| Palen  2006 [71]  US  RCT | HMO (n=16 primary care clinics)  *Ambulatory care* | Provider – Internal medicine and family practice physicians (n=207).  Patients – Received prescription for targeted medication (n=26,586). | Non-intrusive alerts. List of medications in CPOE system. Physician orders drug and for selected medications screen automatically includes reminder that laboratory test is required.  Plus usual CPOE and academic detailing.  *System initiated*  *Multi-faceted intervention* | Usual care (CPOE and custom formulary) | 8 | *↑ Laboratory monitoring rates for:* |  |
|  |  |  |  |  |  | Isoniazid (ALT or AST) (n=69) | – (NS) |
|  |  |  |  |  |  | Rifampin (ALT or AST) (n=13) | – (NS) |
| **Anticoagulant Therapy** | | | | | | | |
| Demakis  2000 [21]  US  RCT | Veterans Affairs hospital outpatient centres (n=12)  *Ambulatory care* | Provider – Resident physicians (n=275)  Patients – Eligible for 1 or more standards of care (n=12,989) | Reminder about patients who were suitable for “standards of care review”. Rationale for standard also provided with reminder.  Paper version also provided plus didactic group lecture, written materials.  *System initiated*  *Multi-faceted intervention* | Usual care  Plus didactic group lecture, written materials | 9 | Adherence to monitoring warfarin treatment (every 45 days) (n=563) | + (NS) |
| **Elderly** | | | | | | | |
| Judge  2006 [59]  US  RCT | Long-term care units (n=7)  *Institutional care* | Provider – Physicians, nurse practitioners, physician assistants (n=27).  Patients – Residents of long-term care units (n=4,282 alerts). | Alert generated when entering drug orders if order involved: high-severity drug interactions; was for patient with abnormal laboratory result; monitoring for potential adverse effects; prophylactic measures to address potential adverse effects; dose ranges to reduce adverse effects in the elderly (41 different alerts in total). Alerts included instructions for laboratory monitoring and recommendations for reconsidering drug orders and monitoring for possible side-effects.  Plus usual CPOE.  *System initiated*  *CDSS only* | Usual care (CPOE) | 8 | Appropriate action taken for:  Thiazide or loop diuretics (potassium) (n=411) | – (NS) |
|  |  |  |  |  |  | Phenytoin orders (monitor drug level) (n=21) | – (NS) |
| **Other Clinical Areas** | | | | | | | |
| Feldstein  2006 [31]  US  RCT | HMO (n=15 primary care clinics)  *Ambulatory care* | Provider – Adult medicine primary care providers (n=200).  Patients – Age>18 years, received a new prescription and had not had baseline laboratory monitoring within 5 days after dispensing (n=961). | Electronic message sent to provider from chair of patient safety committee. Message indicated new medication had been dispensed and patient had not received laboratory monitoring. Message referenced internal and external guideline resources, recommended specific tests, and provided sample letter provider could send to patient requesting laboratory test. Reminder sent 9-10 days later if required. Guidelines posted on intranet.  *System initiated*  *Multi-faceted intervention* | Usual care  Plus online guidelines | 10 | Completed laboratory monitoring within 9 days (e.g. lab testing for patients on ACE inhibitors, allopurinol, statins) | **++** |
|  |  |  |  |  |  | Completed laboratory monitoring within 25 days | **++** |
| Koide  2000 [32]  Japan  Quasi-experimental | Hospital inpatient and outpatient  *Ambulatory care*  *Institutional care* | Provider – Physicians (n=37 pre-intervention, n=31 post-intervention).  Patients – Psoriasis (n=54 pre-intervention, n=57 post-intervention). | Physician alerted when prescribing etretinate without patient having ALT or AST test in previous 90 days or had abnormal ALT or AST results. Physician could not ignore alert and accepted it by cancelling prescription or ordering ALT or AST test.  Plus usual CPOE.  *System initiated*  *CDSS only* | Usual care (CPOE) | 4 | Appropriate etretinate for patients with normal ALT or AST | **++** |
| Overhage  1997 [33]  US  RCT | Hospital inpatient (n=6 general medicine services)  *Institutional care* | Provider – Physicians (internists, senior residents, interns; n=86).  Patients – Inpatients (n=2,181) | Rule-based reminder program analysed patients’ EMR to determine if any the corollary orders should be presented when 76 target drugs and 11 tests were ordered. When physician ordered drug a rule-based reminder program analysed data in EMR and determined if any corollary order should be presented. Physician able to accept, reject or modify order.  Plus usual CPOE and written guidelines .  *System initiated*  *Multi-faceted intervention* | Usual care (CPOE)  Plus written guidelines | 10 | Immediate compliance with suggestion (e.g. lab testing for heparin, warfarin, digoxin) | **++** |
|  |  |  |  |  |  | 24 hour and length of hospital stay compliance with suggestion | **++** |
| Palen  2006 [71]  US  RCT | HMO (n=16 primary care clinics)  *Ambulatory care* | Provider – Internal medicine and family practice physicians (n=207).  Patients – Received prescription for targeted medication (n=26,586). | Non-intrusive alerts. List of medications in CPOE system. Physician orders drug and for selected medications screen automatically includes reminder that laboratory test is required.  Plus usual CPOE and academic detailing.  *System initiated*  *Multi-faceted intervention* | Usual care (CPOE and custom formulary) | 8 | *↑ Laboratory monitoring rates for:* |  |
|  |  |  |  |  |  | Methotrexate (CBC, creatinine, ALT or AST, LFT) (n=16) | **++** |
|  |  |  |  |  |  | Colchicine (CBC) (n=811) | + (NS) |
|  |  |  |  |  |  | Metformin hydrochloride (creatinine) (n=2,038) | – (NS) |
|  |  |  |  |  |  | Phenytoin sodium (ALT or AST, drug levels) (n=135) | + (NS) |
|  |  |  |  |  |  | Carbamazepine (TSH, ALT, CBC, LFT, platelets) (n=272) | – (NS) |
|  |  |  |  |  |  | Valproic acid (ALT or AST, CBC) (n=155) | – (NS) |
|  |  |  |  |  |  | Allopurinol (creatinine) (n=784) | – (NS) |
|  |  |  |  |  |  | Overall compliance with laboratory monitoring suggestion | – (NS) |
| Safran  1995 [22]  US  RCT | Hospital-based general medicine practices (n=5)  *Ambulatory care* | Provider – Resident and staff physicians (n=126), nurse practitioners (n=10).  Patients – HIV (n=349) | Alerts sent automatically to provider about important event (e.g. lab results out of normal range) and reminder posted in patient EMR (e.g. vaccination due). Providers could act upon the alert, indicate alert was inappropriate or not applicable, or indicate patient refused recommendation.  *System initiated*  *CDSS only* | Usual care (access to library and CME seminars) | 9 | Compliance with laboratory testing for zidovudine (CBC) (n=259) | **++** |
| **Dose Calculators** | | | | | | | |
| **Anticoagulant Therapy** | | | | | | | |
| Ageno  2000 [63]  Canada  RCT | Hospital inpatient  *Institutional care* | Provider – Physicians, nurses  Patient – All patients eligible unless INR≥1.4 before treatment (n=101) | Calculated warfarin dose and interval to next appointment based on previously established INR inputted by physician.  *User initiated*  *CDSS only* | Manual dosing by physician | 7 | ↓ INRs above therapeutic range | + (NS) |
|  |  |  |  |  |  | ↓ Duration of therapy | + (NS) |
|  |  |  |  |  |  | ↓ Dose adjustments | – (NS) |
| Ageno  1998 [62]  Canada  RCT | Hospital outpatient  *Institutional care* | Provider – 3 nurses and 2 physicians in comparison group. No other information.  Patients – Long-term anticoagulant therapy after valve replacement surgery (n=101) | Calculated warfarin dose and interval to next appointment based on previously established INR inputted by physician.  *User initiated*  *CDSS only* | Manual dosing by physician | 8 | ↓ Dose adjustments | **++** |
|  |  |  |  |  |  | ↑ Time interval between blood tests | + (NS) |
|  |  |  |  |  |  | ↓ Blood tests required | + (NS) |
|  |  |  |  |  |  | ↑ Time spent in therapeutic INR range (203 to 3.7) | + (NS) |
|  |  |  |  |  |  | ↑ INRs in therapeutic range (2.3 to 3.7) | + (NS) |
| Fihn  1994 [66]  US  RCT | Hospital outpatient (anticoagulation clinics) (n=5)  *Ambulatory care* | Providers – Physicians  Patients – Planned duration of anticoagulation≥ 6 weeks (n=620) | Algorithm calculating interval to next appointment using patient PTR, target PTR, prior visits, variability in PTR, and costs. Data inputted at each visit. Physician able to modify suggestions.  *User initiated*  *CDSS only* | Usual care | 8 | ↑Time interval between visits | **++** |
|  |  |  |  |  |  | Dose adjustments | + (NS) |
|  |  |  |  |  |  | ↓ Deviation of INR from therapeutic range | – (NS) |
| Manotti  2001 [68]  Italy  RCT | Hospital outpatient (n=5)  *Ambulatory care* | Provider – Physicians  Patients – Stabilisation group: within first 3 months of commencing anticoagulation. Maintenance group: long-term anticoagulant therapy (n=1,251) | Algorithm based on prothrombin times. Database set up with individual patient records. At end of routine procedures generates a personalised prescription containing INR results, dosing and follow-up information.  *System initiated*  *CDSS only* | Manual dosing by physician | 8 | ↓ Number of visits | **++** |
|  |  |  |  |  |  | Time spent in therapeutic INR range | **++** |
|  |  |  |  |  |  | Stabilised (≥3 INR values within therapeutic range a week apart) within first month | **++** |
|  |  |  |  |  |  | Stabilised (≥3 INR values within therapeutic range a week apart) within 2 months | **++** |
| Mitra  2005 [70]  US  RCT | Hospital inpatient  *Institutional care* | Provider – Physicians  Patients – Length of stay>2 weeks, no complications, target INR>2 (n=30) | System calculated warfarin dose, timing and frequency of next blood test after physician inputted patient INR. Physician free to accept or modify suggestions.  *User initiated*  *CDSS only* | Manual dosing by physician | 8 | Time spent in therapeutic INR range | **++** |
|  |  |  |  |  |  | ↓ Number of blood tests | – (NS) |
| Poller  1993 [72]  UK  RCT | Hospital outpatient (anticoagulation clinics)  *Ambulatory care* | Provider – Physicians  Patients – Recently discharged from hospital and long-term anticoagulant therapy >6 months (n=186) | System calculated warfarin dose and interval to next appointment after input of INR.  *User initiated*  *CDSS only* | Manual dosing by physician | 8 | INRs in therapeutic range (INR target 3.0-4.5) | **++** |
|  |  |  |  |  |  | INRs in therapeutic range (INR target 2.0-3.0) | – (NS) |
|  |  |  |  |  |  | ↓ Time interval between visits | + (NS) |
| Poller  1998 [73]  UK, Norway, Denmark, Portugal  RCT | Hospital outpatient  *Ambulatory care* | Provider – Physicians  Patients – Stabilisation group: discharged from hospital within 6 weeks of commencing anticoagulation. Stable group: long-term anticoagulant therapy (n=254) | System calculated warfarin dose and interval to next appointment after input of INR.  *User initiated*  *CDSS only* | Manual dosing by physician | 5 | More time spent in therapeutic INR range | **++** |
|  |  |  |  |  |  | ↓ INRs not in therapeutic range | + (NS) |
|  |  |  |  |  |  | ↑ Time interval between visits | + (NS) |
|  |  |  |  |  |  | ↓ Dose adjustments | + (NS) |
| Vadher  1997 [74]  UK  RCT | Hospital inpatient and outpatient  *Ambulatory care*  *Institutional care* | Provider – Trainee physicians (n=48), nurse practitioner (n=1)  Patients – Eligible for the initiation of warfarin (n=148) | System calculated induction dose and interval to next INR measurement. Physicians could reject the recommendations.  Plus written guidelines.  *System initiated*  *Multi-faceted intervention* | Manual dosing by physician  Plus written guidelines | 8 | ↑ Time to first pseudo-event (over or under anticoagulation) | **++** |
|  |  |  |  |  |  | ↓ Time to reach stable dose | **++** |
|  |  |  |  |  |  | ↑ Time spent in therapeutic INR range | + (NS) |
|  |  |  |  |  |  | Time to reach therapeutic range | 0 |
|  |  |  |  |  |  | Time interval between blood tests | 0 |
| **Antibiotics** | | | | | | | |
| Burton  1991 [64]  US  RCT | Medical and surgical wards of Veterans Affairs hospital (n=17 teams)  *Institutional care* | Provider – Physicians  Patients – Admitted with infection, treatment for at least 48 hours, no history of toxicity, adequate renal function (n=147) | System calculated aminoglycoside dose after data input using predictive algorithm with subsequent adjustments based on peak and trough serum aminoglycoside concentrations.  *User initiated*  *CDSS only* | Manual dosing by physician | 7 | ↑ Peak serum aminoglycoside concentration | **++** |
|  |  |  |  |  |  | ↓ Duration of aminoglycoside therapy | + (NS) |
|  |  |  |  |  |  | ↓ Incidence of aminoglycoside toxicity | + (NS) |
| **Respiratory** | | | | | | | |
| Casner  1993 [65]  US  RCT | Hospital inpatient  *Institutional care* | Provider – Physicians  Patients – Age>18 years with asthma or COPD (n=35) | Program calculated dosing regimen after data input to achieve target theophylline level. Calculations based on 2 serum theophylline measurements.  *User initiated*  *CDSS only* | Manual dosing by physician | 5 | ↑ Therapeutic levels of theophylline | + (NS) |
|  |  |  |  |  |  | ↓ Toxic levels of theophylline | – (NS) |
|  |  |  |  |  |  | ↓ Sub-therapeutic levels of theophylline | – (NS) |
|  |  |  |  |  |  | ↓ Theophylline associated episodes of toxicity | – (NS) |
|  |  |  |  |  |  | ↓ Days of theophylline administration | – (NS) |
| Verner  1992 [75]  Israel  RCT | Hospital inpatients  *Institutional care* | Provider – Physicians  Patients – Presented to emergency with acute bronchospasm. Entered study if IV theophylline had not been given and salbutamol nebuliser did not relieve symptoms (n=25) | Program calculated dosing regimen after data input to achieve target theophylline level.  *User initiated*  *CDSS only* | Manual dosing by physician | 6 | ↑ Theophylline levels in therapeutic range | **++** |
| **Other Clinical Areas** | | | | | | | |
| Lesourd  2002 [35]  France  RCT | Hospital outpatients (n=3)  *Ambulatory care* | Providers – Physicians  Patients – Women undergoing ovarian stimulation for infertility (n=164) | Treatment recommendations. Provider enters patient data (e.g. duration and type of infertility, FSH, LH). System evaluates ovary response and gives dose recommendations. Physician makes further measurements and enters data then software proposes dosing. Cancellation of cycle may be recommended.  *User initiated*  *CDSS only* | Usual care (monitored by physician) | 6 | Appropriate number of FSH units administered | U |
|  |  |  |  |  |  | Duration of stimulation | 0 |

* Unless otherwise stated, number of patients is close to or equal to that specified in the “participants” column, or was not reported.

+ (NS) indicates intervention favoured the CDSS but was not statistically significant; – (NS) indicates intervention favoured comparison group but was not statistically significant; 0 = no difference between groups; **++** indicates intervention favoured CDSS and was statistically significant; **- -** indicates intervention favoured comparator and was statistically significant; U = unclear.

ACE = angiotensin-converting enzyme; BP = blood pressure; ALT = alanine aminotransferase; AST = aspartate aminotransferase; CBC = complete blood count; CDSS = computerised clinical decision support system; CHD = coronary heart disease; CME = continuing medical education; CPOE = computerised provider order entry; COPD = chronic obstructive pulmonary disease; EMR = electronic medical record; FSH = follicle-stimulating hormone; HIV = human immuno-deficiency virus; HMO = health maintenance organisation; INR = international normalised ratio; IV = intravenous; LFT = liver function test; LH = luteinizing hormone; NYHA = New York Heart Association; PTR = prothrombin time ratio; RCT = randomised controlled trial; TSH = thyroid stimulating hormone;
