# Supplementary material for: Do computerised clinical decision support systems for prescribing change practice? A systematic review of the literature (1990-2007)
Source: BMC Health Serv Res. 2009 Aug 28;9:154. doi: 10.1186/1472-6963-9-154 (PMC2744674; doi:10.1186/1472-6963-9-154)
Supplement: Additional file 7 — Table S6: Key study features and results (Stopping treatment). * Unless otherwise stated, number of patients is close to or equal to that specified in the "participants" column, or was not reported. + (NS) indicates intervention favoured the CDSS but was not statistically significant; - (NS) indicates intervention favoured comparison group but was not statistically significant; 0 = no difference between groups; ++ indicates intervention favoured CDSS and was statistically significant; - - indicates intervention favoured comparator and was statistically significant; U = unclear. CDSS = computerised clinical decision support system; CPOE = computerised provider order entry; COPD = chronic obstructive pulmonary disease; EMR = electronic medical record; FSH = follicle-stimulating hormone; GP = general practitioner; LH = luteinizing hormone; NSAID = non-steroidal anti-inflammatory drugs; NYHA = New York Heart Association; RCT = randomised controlled trial. [file 1472-6963-9-154-S7.doc]

**Table V: Key study features and results (Stopping treatment)**

| **Study** | **Setting** | **Participants** | **Interventions** | **Comparison Group** | **QA Score (N/10)** | **Practice change in line with intent of CDSS *** | **Change in performance** |
| --- | --- | --- | --- | --- | --- | --- | --- |
| Lesourd  2002 [35]  France  RCT | Hospital outpatients  *Ambulatory care* | Providers – Physicians  Patients – Women undergoing ovarian stimulation for infertility (n=164) | Treatment recommendations. Provider enters patient data (e.g. duration and type of infertility, FSH, LH). System evaluates ovary response and gives dose recommendations. Physician makes further measurements and enters data then software proposes dosing. Cancellation of cycle may be recommended.  *User initiated*  *CDSS only* | Usual care (monitored by physician) | 6 | Number of cancelled cycles | U |
| Tamblyn  2003 [36]  Canada  RCT | General practice  *Ambulatory care* | Provider – GPs age>30 years with minimum of 100 elderly patients and 70% of time fee-for-service (n=107)  Patients – Age>66 years (n=12,560) | Alert identified 159 clinically relevant prescribing problems in the elderly (drug-disease contraindications, drug interactions, drug-age contraindications, duration of therapy, therapeutic duplication). Alerts appeared when patients’ EMR was opened, prescription records downloaded, and when physician updated EMR with patients’ health problem and prescriptions. Alert identified the nature of the problem, possible consequences and alternatives.  *System initiated*  *CDSS only* | Usual care | 9 | Discontinuation of inappropriate prescriptions (e.g. NSAIDs, benzodiazepines) (n=3,248) | + (NS) |
|  |  |  |  |  |  | ↓ Excess duration of therapy (n=1,054) | + (NS) |
|  |  |  |  |  |  | ↓ Drug-disease contraindication (n=1,814) | + (NS) |
|  |  |  |  |  |  | ↓ Drug interaction (n=282) | + (NS) |
|  |  |  |  |  |  | ↓ Drug-age contraindication (n=1,448) | – (NS) |
|  |  |  |  |  |  | ↓ Therapeutic duplication (n=326) | – (NS) |
| Tierney  2005 [34]  US  RCT | Hospital-based general internal medicine practices (n=4)  *Ambulatory care* | Provider – Physicians (general internists, internal medicine, medicine-paediatric residents; n=274), pharmacists (n=20).  Patients – Age≥18 years with asthma, COPD, emphysema or had received ≥2 respiratory medications (n=706). | Care suggestions for asthma and COPD management. Suggestions based on data from patient EMR and data entered by physician (vital signs, symptoms, NYHA class). Computer screen displayed suggested order, possible actions and brief explanation. Physicians could view guidelines and references via “help” key.  Plus usual CPOE with alerts, written guidelines, didactic group and one-on-one lectures.  *Mixed*  *Multi-faceted intervention* | Usual care (CPOE with alerts)  Plus written guidelines, didactic group and one-on-one lectures | 10 | Stop ipratropium (n=43) | – (NS) |

* Unless otherwise stated, number of patients is close to or equal to that specified in the “participants” column, or was not reported.

+ (NS) indicates intervention favoured the CDSS but was not statistically significant; – (NS) indicates intervention favoured comparison group but was not statistically significant; 0 = no difference between groups; **++** indicates intervention favoured CDSS and was statistically significant; **- -** indicates intervention favoured comparator and was statistically significant; U = unclear.

CDSS = computerised clinical decision support system; CPOE = computerised provider order entry; COPD = chronic obstructive pulmonary disease; EMR = electronic medical record; FSH = follicle-stimulating hormone; GP = general practitioner; LH = luteinizing hormone; NSAID = non-steroidal anti-inflammatory drugs; NYHA = New York Heart Association; RCT = randomised controlled trial.
