# Supplementary material for: Do computerised clinical decision support systems for prescribing change practice? A systematic review of the literature (1990-2007)
Source: BMC Health Serv Res. 2009 Aug 28;9:154. doi: 10.1186/1472-6963-9-154 (PMC2744674; doi:10.1186/1472-6963-9-154)
Supplement: Additional file 8 — Table S7: Key differences between the studies included in the current review and previous reviews. * Studies were not prescribing focused (screening, preventive care/disease management). † Studies addressed automated drug dosing studies without decision support. # Review excluded studies below a defined quality rating. ‡ Review did not include monitoring or ceasing studies. [file 1472-6963-9-154-S8.doc]

**Table VI: Key differences between the studies included in the current review and previous reviews**

|  | **Garg et al ^1^** | **Kawamoto et al^2^** | **Mollon et al^9^** | **Durieux et al^5^** |
| --- | --- | --- | --- | --- |
| 1. Total studies | 100 | 88 | 41 | 23 |
| 1. Studies in common with current review | 27 | 7 | 22 | 8 |
| 1. Studies in their review but not in current review (#1 minus #2) | 73 | 81 | 19 | 15 |
| a. Studies identified in current literature search but excluded from analysis (not prescribing, not physicians, no prescribing data, no prescribing outcome, not electronic, insufficient information, weak quasi-experiments) | 30 | 26 | 14 | 5 |
| b. Studies outside search dates of current review | 30 | 25 | 5 | 7 |
| c. Studies within search dates but not identified in current literature search | 13* | 30* | - | 3^†^ |
| 1. Studies in current review but not in other reviews (56 minus #2) | 29 | 49 | 34 | 48 |
| a. Studies in current review not meeting inclusion criteria of other reviews | 5 | 24^#^ | 34^‡^ | 45 |
| b. Studies in current review published after cut-off dates of other reviews | 24 | 25 | - | 3 |

* Studies were not prescribing focused (screening, preventive care/disease management)

† Studies addressed automated drug dosing studies without decision support

# Review excluded studies below a defined quality rating

‡ Review did not include monitoring or ceasing studies
